# Strange semimetal dynamics in SrIrO_3_

**DOI:** 10.1038/s41467-020-18092-6

**Published:** 2020-08-26

**Authors:** K. Sen, D. Fuchs, R. Heid, K. Kleindienst, K. Wolff, J. Schmalian, M. Le Tacon

**Affiliations:** 1 Institut für Quantenmaterialien und -technologien, Karlsruher Institut für Technologie, 76021 Karlsruhe, Germany; 2 Institut für Theorie der Kondensierten Materie, Karlsruher Institut für Technologie, 76131 Karlsruhe, Germany

**Keywords:** Electronic properties and materials, Surfaces, interfaces and thin films

## Abstract

The interplay of electronic correlations, multi-orbital excitations, and spin-orbit coupling is a fertile ground for new states of matter in quantum materials. Here, we report on a polarized Raman scattering study of semimetallic SrIrO_3_. The momentum-space selectivity of Raman scattering allows to circumvent the challenge to resolve the dynamics of charges with very different mobilities. The Raman responses of both holes and electrons display an electronic continuum extending far beyond the energies allowed in a regular Fermi liquid. Analyzing this response within a memory function formalism, we extract their frequency dependent scattering rate and mass enhancement, from which we determine their DC-mobilities and electrical resistivities that agree well with transport measurement. We demonstrate that its charge dynamics is well described by a marginal Fermi liquid phenomenology, with a scattering rate close to the Planckian limit. This demonstrates the potential of this approach to investigate the charge dynamics in multi-band systems.

## Abstract (web summary)

It remains challenging to resolve the dynamics of charges with different mobilities in multi-band systems. Here, the authors report a Raman scattering study of the dynamics of holes and electrons in semimetallic SrIrO_3_, which is well described by a marginal Fermi liquid phenomenology, with frequency dependent scattering rates close to the Planckian limit.

## Introduction

Numerous quantum materials have exotic electronic properties that cannot be accounted for within the canonical framework of the Fermi-liquid theory. They attract increasing attention both because of the profound challenge they pose to our fundamental understanding of electrons in condensed matter^1^ and because of their technological potential^2^. Over the past decade, the spin–orbit coupling (SOC), which describes the strength of the interaction between the spin and the orbital motion of a quasiparticle, has been identified as one of the major ingredient for the realization of novel quantum phases of matter^3^, encompassing in particular topological or axion insulators, and quantum spin liquids, as well as Dirac or Weyl topological semimetals. The interplay between electronic correlations and semimetal behavior has, for example, been discussed in the context of quantum criticality in Dirac systems^4,5^ and topological and non-Fermi-liquid states that emerge near quadratic band touching points^6^.

The 5*d* transition metal oxides are particularly interesting materials in this respect. In the iridate perovskites from the Ruddlesden–Popper series Sr_*n* + 1_Ir_*n*_O_3*n* + 1_, the crystal field lifts the degeneracy of the 5*d* levels of the octahedrally coordinated Ir^4+^ ions. Combined with electron–electron correlations (*U* ~ 2 eV) and large SOC (~0.4 eV), this yields a peculiar type of insulating antiferromagnetic phases at half-filling in Sr_2_IrO_4_ (*n* = 1) and Sr_3_Ir_2_O_7_ (*n* = 2) compounds. This phase is known as the spin–orbital Mott state^7^, in which *J*_eff_ = 1/2 pseudo-spins rather than pure spins order magnetically and is widely seen as a novel platform for unconventional superconducting states. In addition, a plethora of fascinating exotic physical phenomena, encompassing pseudogap or Fermi arc states, have been discovered in these systems and remain to be understood^8^. The *n* = *∞* phase of this series, SrIrO_3_ (SIO), exhibits, on the other hand, a semimetallic and paramagnetic ground state^9–11^, and has long been predicted to host Dirac quasiparticles near the Fermi energy *E*_F_^12,13^, making it a potential realization of a correlated Dirac semimetal^14^.

Recent angle-resolved photoemission spectroscopy (ARPES) studies on SIO directly confirmed the semimetallic character of this compound, in which both a heavy hole-like and a lighter electron-like band cross the Fermi level *E*_F_^15,16^, as predicted by first-principles calculations^12,13,17^. Despite a very steep and quasi-linear dispersion of the electron-like band, the theoretically predicted^12,13^ symmetry-protected degeneracy of the Dirac point has been found to be lifted^16^. Although evidences for the proximity of SIO to a magnetic insulating state have been reported^18–20^, the sizeable mixing of the *J*_eff_ = 1/2 and 3/2 character of the narrow bands crossing *E*_F_ strongly contrasts with the case of Sr_2_IrO_4_.

Significant efforts have been devoted to understand the charge dynamics in this system. However, disentangling the contribution of both types of carriers in such multiband systems is generally a delicate task using conventional transport methods. In the particular case of SIO, since the effective mass of quasi-linearly dispersing electrons is much smaller than the one of the heavier holes^15^, the electrons have a higher mobility (*μ* = *eτ*_0_/m*, *τ*_0_: static relaxation time) and dominate Hall effect measurements^11,21,22^. Another study suggests that the electrical conductivity and thermopower are affected by both electron- and hole-like carriers^23^. Both types of charge carriers further contribute to the optical conductivity^11,24,25^.

Consequently, and to the best of our knowledge, neither the static nor the dynamical scattering rates and mass enhancements of the electron- and hole-like carriers were determined experimentally in SIO. Investigations of the charge dynamics in this system are further complicated by the fact that, unlike the *n* = 1 and 2 compounds from which single crystals can be grown, the perovskite phase of SIO is metastable and can only be synthesized in polycrystalline or in thin film forms. Subtle structural differences between the polycrystalline materials and the thin films can yield different behavior at low temperatures (electronic structure reconstruction, location of the Dirac node, metal-to-insulator transition, sign of the magnetoresistance, etc.) and generally illustrate the strong sensitivity of the electronic properties of this system to structural details^20,26^. Even for a given substrate, slight changes in the lattice parameters can induce metal-to-insulator transition^27^. For most of the substrates, significant lattice mismatches induce large strain effects, as well as, for example, twin or grain boundaries that considerably affect the electrodynamics of the system. The Hall coefficient, the carrier density, or the resistivity have recently been reported to be strongly substrate and film thickness dependent^18,22,28,29^, and also indicate strong strain–relaxation effects. As both first-principles calculations^12,13^ and ARPES^15,16^ investigations have shown that the hole and electron pockets crossing the Fermi level occupy distinct regions of the reciprocal space, the way towards momentum-selective studies of the charge dynamics^30–32^ in SIO via electronic Raman scattering (ERS) is paved.

In this communication, we take full advantage of the polarization selection rules of ERS to independently investigate the intrinsic charge dynamics of the hole- and electron-like carriers in a fully strain-relaxed 50-nm-thick SIO thin film. Exploiting the confocality of our micro-Raman setup, we have been able to extract the electronic response from the thin film and to reveal the existence, for both types of charge carriers, of a flat electronic continuum extending at least up to 1000 cm^−1^ (~125 meV). This is strongly reminiscent of the Raman response of doped high-*T*_c_ superconducting cuprates^33–35^. This in turn suggests that such continuum might be a universal feature of correlated electron system on the verge of a Mott transition. The reported electronic continuum is a characteristic feature of the marginal Fermi-liquid (MFL) phenomenology^36^ and has been analyzed using a memory function formalism, which allowed us in particular to quantitatively extract charge-carrier-resolved frequency-dependent scattering rates Γ(*ω*, *T*) and mass enhancements m*/*m*_b_ = 1 + *λ*(*ω*, *T*), where m* and *m*_b_ are the effective mass renormalized by electron–electron (e–e) interactions and the bare band mass, respectively. For both type of carriers, these quantities amount for a rather broad temperature regime to an inverse quasiparticle time

1 $$\hslash {\tau }^{-1}=\Gamma /(1+\lambda ),$$

which is surprisingly close to the Planckian limit^37,38^${\tau }_{\hslash }^{-1}={k}_{{\rm{B}}}T/\hslash$ (even though at lowest *T* the MFL theory yields ${\tau }^{-1}\propto T/\mathrm{log}\,(D/T)$, where *D* is an appropriate cut-off frequency as discussed in Supplementary Note 2). More generally, this work demonstrates the potential of polarized ERS for the study of scattering rate, mass enhancement, and mobility of charge carriers in semimetals and other multiband systems.

## Results

### Samples

**Fig. 1:**
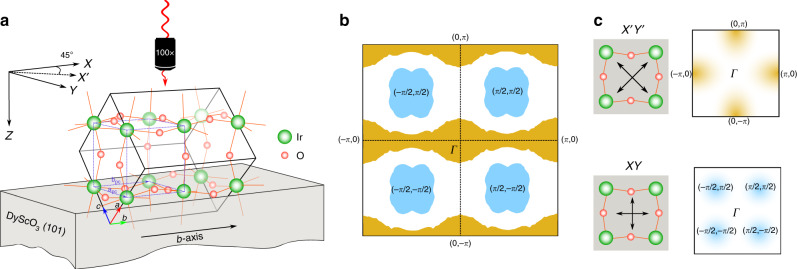
Polarization selection rules. **a** Sketch illustrating orientation of SrIrO_3_ crystal unit cell of *Pnma* space group on (101)-DyScO_3_ substrate. A pseudocubic unit cell of SrIrO_3_ is designated by blue dashed lines. The *XYZ*-coordinate system concerning the backscattering Raman experiments is marked in black arrows. Incoming laser propagates along *Z*-axis, as shown by a red wavy-arrow through a microscope objective. **b** Calculated Fermi surface is shown in 1-Ir (pseudocubic unit cell) Brillouin zone (BZ) for the respective pseudocubic unit cell. The electron pockets are at (±*π*/2, ±*π*/2), whereas the rest arises from hole-like bands. Note that the hole pocket at the Γ-point and at the 1-Ir BZ boundary at (±*π*, 0) are equivalent. **c** Electronic Raman scattering structure factors are shown on 1-Ir BZ (right panels) for the respective scattering configurations, namely ${{Z}}({{X}}^{\prime} {{Y}}^{\prime} )\bar{Z}$ and ${{Z}}({{XY}})\bar{Z}$, as depicted in left panels. The corresponding symmetries are E_*g*_ and T_2*g*_ according to the point group of O_*h*_, respectively.

Epitaxial thin films of SrIrO_3_ (50 nm) were grown by pulsed laser deposition on orthorhombic (*Pnma*) (101)-oriented DyScO_3_ (DSO) substrates and characterized, as described in ref. ^21^. As shown in Fig. 1a, our untwinned films are (101) oriented, with their *b*-axis parallel to the one of DSO substrate. The tilt pattern of the IrO_6_ octahedra, *a*^−^*b*^+^*a*^−^ in Glazer notation is the same as that of bulk SrIrO_3_. The choice of DSO substrates, which has the closest lattice parameters to that of bulk SIO, minimizes epitaxial strain effects. Our recent magnetoresistance measurements on similar DSO-grown SIO films with 9 and 50 nm indicate that the electric transport is already strain independent, at least from the thickness of 9 nm onwards^39^.

### Fermi surface

We have calculated the Fermi surface of the (101)-oriented SIO with respect to its pseudocubic unit cell (UC), in which the Ir atoms sit at the corners of the pseudocube using first-principles calculations, as depicted in Fig. 1b. In agreement with previous reports and ARPES studies^15,16^, we find multiple electron bands crossing the Fermi level around (±*π*/2, ±*π*/2), and several flat hole bands extending along the Γ–*X* directions from the Γ and *Y* points.

### Polarization selection rules

Confocal Raman scattering experiments were carried out in backscattering geometry. As detailed in the “Method” section (and in the Supplementary Figs. 1 and 2), the confocal geometry allows us to accurately separate the intrinsic Raman response of the SIO thin film from that of the DSO substrate^40,41^. Figure 1c summarizes the Raman polarization selection rules relevant for the present study, which allow to selectively probe the charge dynamics on different sections of the Fermi surface of SIO. The *XYZ*-coordinate system used to label the polarization of the light is oriented along the Ir–Ir bonds of the pseudocubic UC (incident laser propagates in the direction perpendicular to the plane of the film, along the *Z*-axis). The Raman vertex will be accordingly described within the cubic O_*h*_ point group. When the incident photon polarization is at 45^∘^ to the Ir–O–Ir bonds and the scattered photons are detected in a crossed-polarization configuration (Porto’s notation: ${{Z}}({{X}}^{\prime} {{Y}}^{\prime} )\bar{{{Z}}}$), the corresponding electronic Raman structure factor has the E_*g*_ symmetry (*x*^2^ − *y*^2^), which probes the dynamics of charge carriers with momentum along the (0, ±*π*) and (±*π*, 0) directions of the 1-Ir Brillouin zone (BZ), as shown in Fig. 1c. Similarly, the electronic Raman structure factor in ${{Z}}({{XY}})\bar{Z}$ has the symmetry of T_2*g*_ (*xy*), which probes the charge carriers whose momentum is located around the (±*π*/2, ±*π*/2) in 1-Ir BZ, as shown in Fig. 1c. Thus, in summary, ${{{X}}}^{\prime}{{{Y}}}^{\prime}$ and *XY* geometries predominantly probe dynamics of hole and electron pockets, respectively.

### Raman responses in *X*′*Y*′ and *XY*

**Fig. 2:**
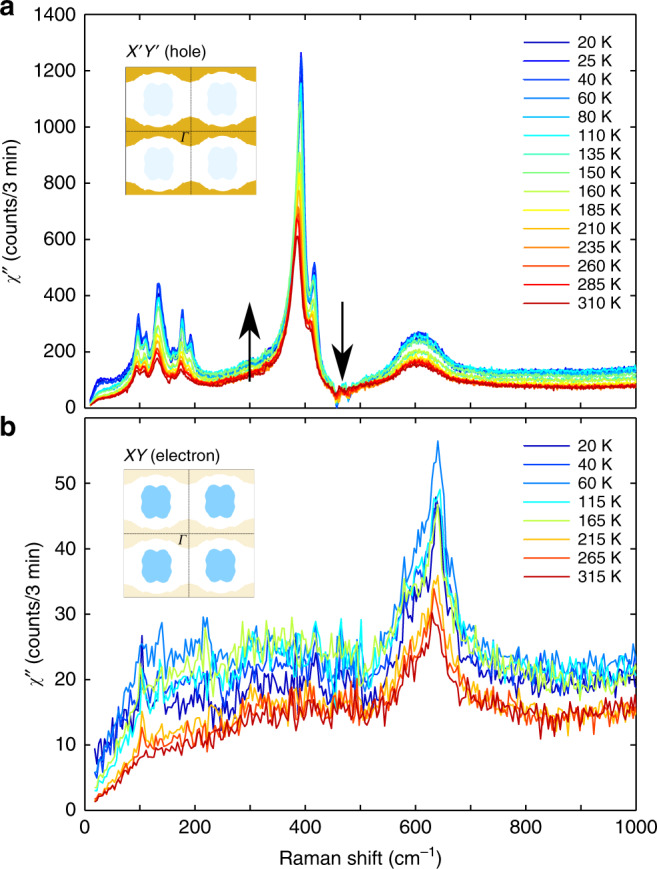
Symmetry-dependent Raman spectra. **a** Raman spectra (*χ″*(*ω*)) in ${{{X}}}^{\prime}{{{Y}}}^{\prime}$ at several temperatures. The arrows indicate spectral weight loss and gain above and below the phonon modes ~400 cm^−1^, respectively. **b** The corresponding plot in *XY* at a few temperatures.

Figure 2a, b display the Raman response *χ″*(*ω*) of the SIO film in ${{{X}}}^{\prime}{{{Y}}}^{\prime}$ and *XY* geometries, respectively, at several temperatures (*T*). In both cases, *χ″*(*ω*) consists of sharp phonons that are superimposed to a flat continuum extending over the whole range of investigated frequencies. Before discussing the origin of this continuum, it is crucial to check that it does not arise from an artifact related to the subtraction of the substrate’s contribution. To do this, we applied the same procedure to a 60-nm-thick film of insulating CeO_2_ (see Supplementary Fig. 3), in which no continuum in the Raman response is expected. Its absence in the experimental spectrum therefore confirms that the featureless Raman response observed in SIO is intrinsic. This continuum is strongly reminiscent of the electronic Raman response encountered in high-*T*_c_ cuprates^30–35^ or Fe-based superconductors^42,43^, in which it arises from the creation of particle–hole excitations across the Fermi level (*E*_F_)^30^. Our interpretation for SIO is supported by the observation that the continuum is ~5 times bigger in ${{{X}}}^{\prime}{{{Y}}}^{\prime}$ than in XY, as qualitatively expected from the larger density of states (DOS) of the flat hole-like bands at (0, ±*π*) and (±*π*, 0) in comparison to the almost linearly dispersing electron-like bands around (±*π*/2, ±*π*/2).

### Phonon contribution

In order to analyze the ${{{X}}}^{\prime}{{{Y}}}^{\prime}$ Raman continuum in details, it is necessary to subtract the other recorded spectral features (Fig. 2a). We observe nine peaks (7 between 90 and 220 cm^−1^, and 2 more ~400 cm^−1^) that sharpen and harden as temperature is lowered and that correspond to Raman active lattice vibrations of SIO. Their detailed analysis will be the subject of a separate study. The broader peak centered around 600 cm^−1^ is only weakly temperature dependent, and its frequency is larger than the calculated cut-off frequency of the phonon spectrum (560 cm^−1^ at the Γ point). We therefore tentatively assess this to a double-phonon process (typically two phonons from the high DOS region of dispersion with opposite momenta) and its contribution to the Raman response can be easily subtracted. A close inspection of the two most intense modes ~400 cm^−1^ reveals that the lower energy one is significantly asymmetric, that is, spectral weight gain toward low frequency seemingly occurs at a cost of spectral weight loss at high frequency (highlighted by arrows in Fig. 2a). This lineshape, known as a Fano resonance^44^, generally arises from the coupling between a discrete excitation and a continuum. Crucially here, this implies that the electronic continuum cannot be analyzed independently and that its coupling to the phonon should explicitly be taken into account (see “Methods”). The contribution of all the other phonon lines can be modeled using regular Lorentzian lineshapes. The analysis in the *XY* channel is comparatively simple, since the corresponding spectra have only a broad feature >600 cm^−1^, which we also attribute to double-phonon scattering. It can be easily subtracted out and that does not distort the underlying *e*-continuum scattering background.

### Electronic Raman scattering

To extract the pure electronic contribution *χ″*_e_(*ω*, *T*) from the ${{{X}}}^{\prime}{{{Y}}}^{\prime}$ data, we follow the approach described in ref. ^45^ and use a phenomenological model (see Eq. (8) in “Methods”) to fit the Raman response *χ″*(*ω*, *T*). Importantly, this approach (described in more details in “Methods”) requires an analytical form for the *χ″*_e_(*ω*, *T*) that we express in terms of a memory function *M*(*ω*, *T*). The method was originally introduced by Götze and Wölfle^46^ to calculate the frequency-dependent optical conductivity of metals, and has been generalized to extract dynamical scattering rate Γ(*ω*, *T*) and mass enhancement factor 1 + *λ*(*ω*, *T*) of charge carriers. It was adapted to analyze ERS in high-*T*_c_ cuprates^47^ and Fe-based superconductors^43^. In the memory function formalism, the experimentally challenging determination of the ERS intensity in absolute units is not required and has not been attempted here. Note that in the following, only electron–electron scattering contribution to the total scattering rate (in particular in the static limit) are included, and possible contributions of long wavelength acoustical phonons to the total scattering rate have not been considered (see Supplementary Note 5 for a detailed discussion).

The memory function parametrisation of the total electronic Raman response function reads^47^:

2 $${\chi }_{e}(\omega ,T)=\frac{M(\omega ,T)}{\hslash \omega +M(\omega ,T)},$$

The real (${\chi }_{e}^{\prime}$) and imaginary (*χ″*_e_) parts of *χ*_e_ are even and odd functions of the Raman shift *ω*, respectively. Thus, we can write

3 $$M(\omega ,T)=\hslash \omega \lambda (\omega ,T)+i\Gamma (\omega ,T),$$

where *λ*(*ω*, *T*) and Γ(*ω*, *T*) are real even functions that are related via a Kramers–Kronig relation.

*χ″*_e_(*ω*, *T*) follows from Eqs. (2) and (3) as

4 $${\chi }_{e}^{^{\prime\prime} }(\omega ,T)=\frac{\hslash \omega \Gamma (\omega ,T)}{{\left[\hslash \omega (1+\lambda (\omega ,T))\right]}^{2}+{\left[\Gamma (\omega ,T)\right]}^{2}}.$$

In analogy to the optical conductivity, we can introduce the quasiparticle scattering time *τ* of Eq. (1), which includes the mass renormalization, contained in the real part of the memory function, that is, in *λ*, see, for example, ref. ^48^.

To proceed, we analyze specific forms of the scattering rate Γ(*ω*, *T*) and determine *λ*(*ω*, *T*) via Kramers–Kronig transformation. Γ(*ω*, *T*) essentially contains two terms, arising from temperature-independent impurity scattering (Γ_imp_) and electron–electron scattering, respectively. For a conventional metal (Fermi liquid), this electron–electron scattering rate is given by:

5 $${\Gamma }_{\mathrm{FL}}(\omega ,T)=\frac{g}{{E}_{\mathrm{F}}}\left[{\left(\hslash \omega \right)}^{2}+{\left(\beta {k}_{\mathrm{B}}T\right)}^{2}\right],$$

where the dimensionless coupling constant *g* characterizes the correlation strength^49^. *β* is a numerical coefficient that determines the relative importance of thermal vs. dynamic excitations for the scattering rate. In the case of a single particle scattering rate holds *β* = *π*, while a quantum Boltzmann analysis for the optical conductivity, a two-particle quantity, yields *β* = 2*π*^50,51^. The analysis of refs. ^52,53^ revealed that, in principle, *π* ≤ *β* < *∞* is possible, depending on the relative strength of elastic and inelastic scattering processes. This was for instance reported for URu_2_Si_2_^54^ and UPt_3_^55^.

**Fig. 3:**
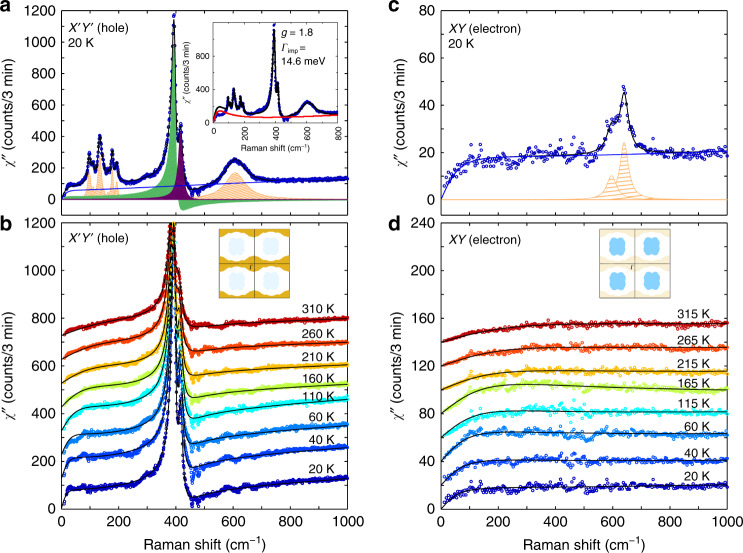
Fits with MFL model to extract electronic Raman scattering response. **a** Raman spectrum at 20 K in ${{{X}}}^{\prime}{{{Y}}}^{\prime}$ (blue circles), with the details of the fit to our phenomenological marginal Fermi-liquid model (solid line in black, see text). The solid line in blue is the best-suited *e*-continuum scattering according to the marginal Fermi-liquid (MFL) model in Eq. (4). Phonon modes are shaded in orange (Lorentzian lineshape) and green or purple (Fano lineshapes). The inset displays a representative attempt to fit these data with Fermi-liquid model. **b** Temperature dependence of the ${{{X}}}^{\prime}{{{Y}}}^{\prime}$ Raman spectra, after subtraction of the symmetrical (Lorentzian) phonon lines. The data were vertically shifted for representation. The solid lines are the best-suited *e*-continuum scattering backgrounds according to the MFL model, including the coupling to the phonons ~400 m^−1^. **c** Raman spectra (*χ″*(*ω*)) in *XY* at 20 K. **d** Temperature dependence of the electronic part or the Raman response in *XY*. The data were vertically shifted for representation. The solid lines are the best-suited *e*-continuum scattering backgrounds according to the MFL model.

The dynamics of both types of charge carriers inferred from our data cannot be adequately described in terms of this Fermi-liquid expression (see inset of Fig. 3a and Supplementary Note 2). This yields our first important conclusion, namely, charge dynamics in SIO is non-Fermi liquid like. To gain further insights, we therefore modeled the Raman response in terms of a singular quasiparticles scattering rate as $\Gamma (\omega ,T)\propto ({\left(\hslash \omega \right)}^{2}+{(\beta {k}_{\mathrm{B}}T)}^{2})^{\alpha }$, with an exponent *α* ≠  1. Acceptable fits of our data can be achieved with values for *α* in the range 0.45 ≤ *α* ≤ 0.6 (see Supplementary Note 2). For simplicity we chose *α*_MFL_ = 1/2, which corresponds to the MFL phenomenology^36^. This choice is also motivated by the fact that the observed featureless ERS continuum of SIO is strongly reminiscent to that of the high-*T*_c_ cuprates^33–35^, in which it is considered as one of the hallmark of the MFL phenomenology^35,36^. Crucially, the dependence of the different fitting parameters on the exact value of *α* lies well within the corresponding error bars. Choosing *α* in the vicinity of *α*_MFL_ will merely change some logarithmic dependencies to power laws with small exponents.

### MFL phenomenology

The characteristics of the MFL is that the quasiparticle weight in the single particle spectrum vanishes like $1/\mathrm{log}\,\left(\frac{D}{\hslash \omega }\right)$, that is, the spectrum tends to be weakly incoherent near the Fermi energy^36^. According to refs. ^35,36^, the scattering rate of the Raman response Γ(*ω*, *T*) for an MFL can be written as

6 $${\Gamma }_{{\rm{MFL}}}(\omega ,T)=g\sqrt{{\left(\hslash \omega \right)}^{2}+{\left(\beta {k}_{\mathrm{B}}T\right)}^{2}},$$

where *g* is again a dimensionless strength of the coupling. Microscopically it is determined by the ratio of the effective e–e correlations, that is, onsite Coulomb repulsion and the bandwidth. *β* plays an analogous role as for the Fermi-liquid rate discussed above.

The total scattering rate is then given by:

7 $$\Gamma (\omega ,T)=\left[{\Gamma }_{{\rm{MFL}}}(\omega ,T)+{\Gamma }_{{\rm{imp}}}\right]\phi (\omega /D),$$

where *ϕ* is an appropriate cut-off function with a cut-off frequency of *D*. We have set *ℏD* to the bandwidth of each charge carrier. In both cases its value amounts to several hundreds of meV, and thereby only weakly affects Γ(*ω*, *T*) in the investigated range of frequencies (see Supplementary Note 2). More details regarding the memory function formalism based on the MFL model are given in the Supplementary Note 2. Representative resulting global fits (including phonon lines and their eventual coupling to the continuum) to *χ″*(*ω*) between *T* = 20 and 310 K for ${{{X}}}^{\prime}{{{Y}}}^{\prime}$ are shown in Fig. 3a and b, respectively. The agreement with the data both at low and high temperatures is excellent and validates the MFL approach to describe the charge dynamics for the hole-like carriers in SIO. We note that the Fano asymmetry parameter for the 391 cm^−1^ mode is essentially temperature independent in the investigated range, indicating no significant change in the electronic structure at the Fermi surface as a function of temperature. This contrasts with the recent optical conductivity report^11^ that has been interpreted as a significant reconstruction of the electron pockets near *E*_F_. We note however that this was observed on polycrystalline samples, with different structural parameters than films, in which these effects are strongly reduced^27^. Similarly, the data recorded in the *XY* channel (Fig. 3c, d) can also be very well fitted in this framework.

### Analysis of Raman spectra with the MFL model

**Fig. 4:**
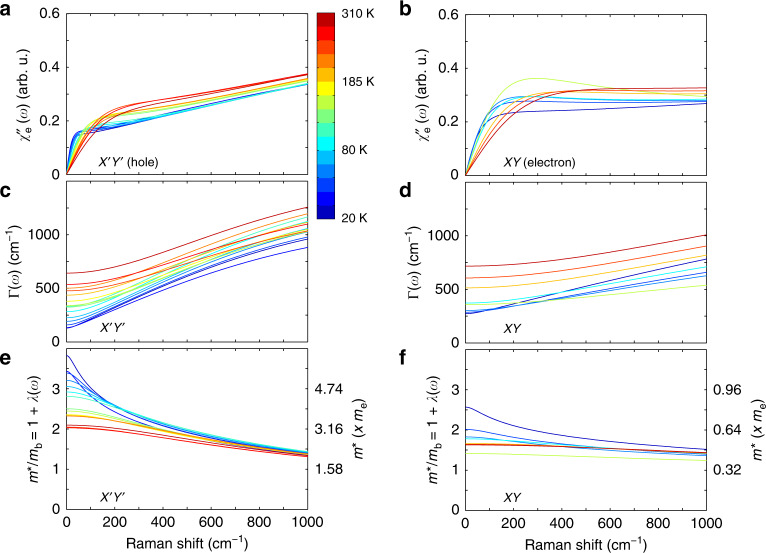
Electronic Raman scattering and charge transport parameters from the MFL fits. Electronic Raman scattering response (*χ″*_e_(*ω*)) at several temperatures in **a** ${{{X}}}^{\prime}{{{Y}}}^{\prime}$ and **b** *XY*. Dynamical scattering rates (Γ(*ω*, *T*)) in **c** ${{{X}}}^{\prime}{{{Y}}}^{\prime}$ and **d** *XY*. The corresponding dynamical mass enhancement factors (m*/*m*_b_(*ω*, *T*)) in **e**, **f**, respectively. They were obtained from the marginal Fermi-liquid (MFL) fits, as shown in Fig. 3.

In Fig. 4a, b, we display the result of our analysis, that is, the pure electronic Raman responses *χ″*_e_(*ω*) in ${{{X}}}^{\prime}{{{Y}}}^{\prime}$ and *XY* channels, respectively. The responses in the two channels appear quite distinct at all temperatures. This is particularly striking at energies *ℏω* ≫ *k*_B_*T*, in which we detect an increase of *χ″*_e_(*ω*) in ${{{X}}}^{\prime}{{{Y}}}^{\prime}$, which, in contrast, remains essentially constant in *XY*. To better understand the origin of such difference, we take a closer look at the values of the parameters *g*, *β*, and Γ_imp_ obtained for each Fermi pockets from our fitting procedure.

First of all, we note that the Γ_imp_ and *β* parameters are both temperature-independent quantities, but both can take very different values for the different Fermi pockets. From our analysis, they respectively amount to ${\Gamma }_{{\rm{imp}}}^{{\rm{hole}}}=12.6\pm 2$ meV and *β*^hole^ = 1.84 ± 0.3 (~0.6*π*) in ${{{X}}}^{\prime}{{{Y}}}^{\prime}$ and ${\Gamma }_{{\rm{imp}}}^{{\rm{elec}}}=30.6\pm 5$ meV and *β*^elec^ = 3.23 ± 0.5 (~*π*) in *XY*.

As shown in Supplementary Fig. 7a, the value of the dimensionless coupling constant *g* (the ratio of the onsite Coulomb repulsion to the bandwidth) is only weakly temperature dependent. It is at least two times larger in ${{{X}}}^{\prime}{{{Y}}}^{\prime}$ (*g* ~ 1.2) than in *XY* (*g* ~ 0.5), indicating stronger e–e correlations on the hole pockets compared to the electron pockets in SIO. This difference can be qualitatively understood as a consequence of the smaller DOS for electron carriers (1.6 states/eV per UC from our calculation) compared to the hole ones (10.7 states/eV per UC), which naturally reduces interaction effects. The values for the electron pockets are similar to those reported for superconducting cuprates YBa_2_Cu_3_O_7_ (*g* =  0.55) and Bi_2_Sr_2_CaCu_2_O_8_ (*g* = 0.4)^35^. Thus, it seems that despite much lower onsite Coulomb repulsion the hole pockets in SIO are effectively at least as correlated as the cuprates’ charge carriers. This is a consequence of the large spin–orbit interaction, which reduces the electronic bandwidth and lowers the critical Coulomb repulsion^56^ required to induce the transition from the current paramagnetic metallic state to the nearby Mott-insulating state^18–20^.

This has a direct impact on the frequency dependence of the dynamical scattering rate Γ(*ω*, *T*) and mass renormalization 1 + *λ*(*ω*, *T*) = m*/*m*_b_(*ω*, *T*) of the two types of carriers. Indeed, the larger *g* in ${{{X}}}^{\prime}{{{Y}}}^{\prime}$ compared to *XY* results in a steeper Γ(*ω*, *T*) for the holes than for the electrons, as shown in Fig. 4c and d, respectively. A larger *g* value also enhances the effective mass of the holes more than that of the electrons (see Fig. 4e, f), yielding an effective mass enhancement ${m}_{0}^{* }/{m}_{\mathrm{b}}=1+{\lambda }_{0}=1+\lambda (\omega \to 0,T)$ of 3.8 for the former against 2.6 for the latter at low temperatures.

### Transport parameters in the static limit

**Fig. 5:**
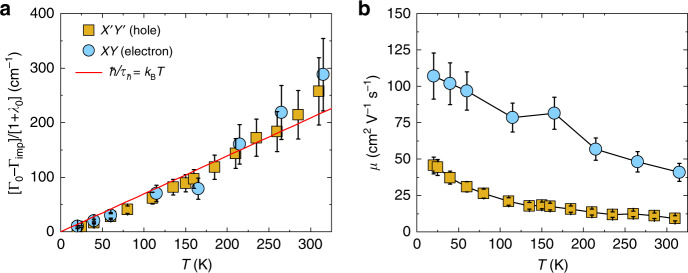
Static charge transport properties. **a** Inverse inelastic quasiparticle scattering time of conduction holes and electrons as function of temperature, both following the universal Planckian limit ($\hslash {\tau }_{\hslash }^{-1}={k}_{\mathrm{B}}T$). **b** Calculated DC mobility for both charge carriers as function of temperature. The error bars reflect the maximum proportional error calculated from the errors on individual fitting parameters (see Supplementary Note 2).

To allow direct comparison with the other experiments, we estimate transport parameters such as the resistivity and mobility of each type of charge carrier from Γ(*ω*, *T*) and 1 + *λ*(*ω* *T*) in the ${{{X}}}^{\prime}{{{Y}}}^{\prime}$ and XY channels in the static limit (*ω* → 0), respectively. Under the assumption that the rates for impurity and inelastic scattering are additive, we obtain for both carrier types that the inverse inelastic quasiparticle time $\hslash {\tau }_{{\rm{inel.}}}^{-1}=({\Gamma }_{0}-{\Gamma }_{{\rm{imp}}})/\left(1+{\lambda }_{0}\right)$ is very close to the Planckian scattering bound^37,38^, *k*_B_*T*, as shown in Fig. 5a. This bound plays an important role in the discussion of transport quantities^48^ where it serves as a measure of the degree of correlation strength of a quantum material. The subsequent comparison of *τ*^−1^ with transport parameters strongly suggests that the rate obtained by Raman scattering allows for the same conclusion. Our observation that *τ*_inel._ ~ *τ*_*ℏ*_ for holes and electrons, characterized by distinct coupling constants *g*, cut-off frequencies *D*, and coefficients *β*, is remarkable. At the lowest temperatures, the logarithmic growth of the MFL mass renormalization yields $\hslash {\tau }^{-1} \sim \frac{\pi \beta }{2}\frac{{k}_{\mathrm{B}}T}{\mathrm{log}\,\frac{D}{\beta T}}$, where holes and electrons reach different quasiparticle rates. In the temperatures range investigated here, however, the MFL mass renormalization remains limited *λ*(*T*) < 3 (see Supplementary Note 2), and the log divergence of *ℏτ*^−1^ is not detectable. At this point, it is unclear whether the observed “universality” of *τ* for different momenta is a coincidence or whether there is a deeper underlying principle at work.

The knowledge of the static quasiparticle scattering time *τ*_0_ = *ℏ*(1 + *λ*_0_)/Γ_0_, together with the assumption that it behaves similar to the one in the electric conductivity, allows for an estimate of the electron mobility $\mu =\frac{e{\tau }_{0}}{{m}_{0}^{* }}$ (*e* is the electronic charge) and of the Drude-like DC resistivity ${\rho }_{0}={m}_{0}^{* }/(n{e}^{2}{\tau }_{0})$ of each type of charge carrier. The former quantity can be directly extracted from our analysis from which we determined the temperature-dependent mass enhancement (with respect to the calculated band masses *m*_b,h_ = 1.58*m*_e_ and *m*_b,e_ = 0.32*m*_e_, see “Methods”) and static rates (Fig. 4), while the latter requires the knowledge of the charge-carrier densities, as discussed below. We summarize the static transport parameters extracted from our analysis at room temperature and 20 K in Table 1, and we plot the extracted mobility of the two charge carriers in Fig. 5b. Despite a larger value of the coupling constant *g* for the holes compared to the electrons, the static relaxation rate Γ_0_(*T*) = *gβk*_B_*T* + Γ_imp_ is comparable for the two types of carriers (see Supplementary Fig. 7b), given the smaller value of *β* and Γ_imp_ for the holes.

**Table 1:** DC mass enhancement factor (m*/*m*_b_), DC scattering rate Γ_0_ = Γ(*ω* → 0), static quasiparticle relaxation time (*τ*_0_ = *ℏ*(1 + *λ*_0_)/Γ_0_), mobility (*μ*), and resistivity (*ρ*_0_) of the conduction holes and electrons around 310 and 20 K.

	${m}_{0}^{* }/{m}_{\mathrm{b}} = 1 + {\lambda}_{0}$	Γ_0_ (cm^−1^)	*τ*_0_ (×10^−15^ s)	*μ* (cm^2^ V^−1^ s^−1^)	*ρ*_0_ (mΩ cm)
Hole (RT)	2.1	642	17.3	9.2	1.4
Electron (RT)	1.6	716	12.1	40.7	0.5
Hole (20 K)	3.8	130	157	45.6	0.3
Electron (20 K)	2.6	273	50	107	0.2

The drastically different mobilities of the two types charge carriers naturally account for the electron-like linear and negative Hall resistance (*R*_*XY*_ vs. *H*) measured on our sample^21^ and previously reported in the literature^15,23^. To detect the field-dependent non-linearities of the Hall resistance arising from the multiband nature of the system, magnetic fields much >15 T would be required (see Supplementary Note 3). This nevertheless implies that the estimate of the charge-carrier density inferred from *R*_*XY*_, assuming that the Hall resistance is only caused by electron carriers, is overestimated. The value obtained for this sample at 2 K, *n*_e_ = 3.0 × 10^20^ cm^−3^ is only weakly temperature dependent (it increases by ~6% at room temperature), and is of the same order of magnitude, albeit larger, than the estimate of ref. ^23^ (*n*_e_ = 1.6 × 10^20^ cm^−3^). Note that the sample of ref. ^23^ was grown on a different substrate, and that the transport properties have been reported to be substrate dependent^22^. Based on this, and on the assumption that *n*_h_ ~ 1.5 × *n*_e_ (as in ref. ^23^), we have estimated the resistivity for each charge carrier. The total resistivity can be calculated by assuming that the two channels conduct current as parallel resistors, yielding *ρ*_0_(20 K) ~0.1 mΩ cm and *ρ*_0_(300 K) ~0.4 mΩ cm. Thus, the value we determine here for the room temperature resistivity of our SIO film is reasonably close to that determined using conventional resistivity measurements (see Supplementary Fig. 9), and is consistent with the values of 0.5–2 mΩ cm reported in the literature^15,18,28,29^. Given, as discussed above, that the Hall measurements tends to overestimate the electron charge-carrier density, both values for the resistivity extracted from the ERS measurements are slightly underestimated, and the agreement with transport is overall very satisfactory at room temperature. The situation is a bit different at low temperature where our transport measurement indicate an upturn in the resistivity, which is not seen here. This upturn is associated with weak localization effects that occur over the length scale probed in the transport measurement, and to which the present experimental approach, which probes the system over a length scale of  ~2 μm (set by the size of our laser spot), is not sensitive. The proposed approach therefore allows us to extract the intrinsic dynamics of the quasiparticles in SIO.

## Conclusion

To summarize, in this work we combined the selection rules of polarized Raman scattering and the high spatial resolution of confocal geometry to investigate independently the dynamics of electron- and hole-like charge carriers in a strain-relaxed film of semimetallic SrIrO_3_ as thin as 50 nm. We find that neither of them can be described within the framework of the Fermi-liquid theory, and that the electronic Raman response can be well modeled using marginal Fermi-liquid (MFL) phenomenology. Using mass enhancement and the DC scattering rate obtained from this analysis allow us to retrieve the mobility for the two types of charge carriers. The results confirmed the much larger mobility of the electron carriers that generally dominate transport experiments. A next natural step would be to investigate how structural degrees of freedom impact this charge dynamics, in particular as SrIrO_3_ is driven across metal-to-insulator and/or magnetic transitions, using for instance lattice strain tuning. The proposed approach more generally demonstrates the power of Raman scattering to resolve the dynamics of charge carriers in correlated semimetals and more generally multiband systems.

## Methods

### Polarization-resolved confocal Raman scattering

Confocal Raman scattering experiments were performed with a Jobin–Yvon LabRAM HR evolution spectrometer in backscattering geometry. We used a He-Ne laser (*λ* = 632.8 nm) with a laser power of ≤0.8 mW that was focused on the sample with a ×100  magnification long-working-distance (7.6 mm) objective. The laser spot size was  ≈2 μm in diameter. The sample was placed on a motorized stage that translate along the incident beam direction which, in combination with a 50 μm confocal hole at the entrance of the spectrometer, provides the high spatial resolution required to investigate thin films. To do so, we first optimized the signal from the film by recording Raman spectra of the sample at different position across the focal point of the microscope. Reference spectra from the substrate was obtained focusing the laser deep in the substrate, and subtracted from the total response (Supplementary Note 1). Spectra were recorded using a grating of 600 grooves/mm, yielding the spectrometer resolution of 1.8 cm^−1^. Any resonance effect in the spectral background was discarded by measuring with a second laser of *λ* = 532 nm. Temperature-dependent measurements were carried out by placing the samples in a He-flow Konti cryostat. All representative spectra were corrected for Bose factors and laser-induced heating of ≤15 K, estimated from systematic study of Raman data with laser power.

### Gobal fit

To disentangle the electronic contribution from the total (electron + phonon) Raman response *Rχ″*(*ω*) in ${{{X}}}^{\prime}{{{Y}}}^{\prime}$ configuration, we fitted the data using the approach proposed by Chen et al.^45^. The Raman response is given by:

8 $$\begin{array}{rcl}R{\chi }^{^{\prime\prime} }(\omega ) = R\left[{\chi }_{e}^{^{\prime\prime} }(\omega )+\mathop{\sum }\limits_{i = 1}^{2}\frac{{g}_{i}^{2}}{{\Lambda }_{i}(\omega )\left[1+{\epsilon }_{i}^{2}(\omega )\right]}\left[{S}_{i}^{2}(\omega )-2{\epsilon }_{i}(\omega ){S}_{i}(\omega ){\chi }_{e}^{^{\prime\prime} }(\omega )-{{\chi }_{e}^{^{\prime\prime} }}^{2}(\omega )\right]\right]\\ {\hskip-96pt}+{\,}R\left[\mathop{\sum }\limits_{j = 1}^{8}\frac{{C}_{j}{w}_{j}}{2\pi }\frac{1}{{\left(\omega -{\omega }_{c,j}\right)}^{2}+{\left(\frac{{w}_{j}}{2}\right)}^{2}}\right],\end{array}$$

where *R* is a proportionality constant, which is a function of temperature in the present case, imaginary part (*χ″*_e_(*ω*)) of the pure electronic Raman response is entangled with two phonon modes through the Fano terms expressed in the second term of Eq. (8), and the last term accounts for the remaining eight phonon modes that have Lorentzian lineshapes. *ϵ*_*i*_(*ω*) and *S*_*i*_(*ω*) are defined as ${\epsilon }_{i}(\omega )=({\omega }^{2}-{\Omega }_{i}^{2})/2{\omega }_{0,i}{\Lambda }_{i}(\omega )$ and ${S}_{i}(\omega )={S}_{0,i}+{\chi }_{e}^{\prime}(\omega )$, respectively. Here, ${\chi }_{e}^{\prime}(\omega )$ is the real part of the electronic Raman response, and was obtained from Eq. (2). Renormalized phonon frequency and linewidth in the presence of electron–phonon coupling (*g*_i_) are defined as ${\Omega }_{i}^{2}={\omega }_{0,i}^{2}-2{\omega }_{0,i}{g}_{i}^{2}{\chi }_{e}^{\prime}(\omega )$ and ${\Lambda }_{i}(\omega )={\Lambda }_{0,i}+{g}_{i}^{2}{\chi }_{e}^{^{\prime\prime} }(\omega )$, respectively. *ω*_0,*i*_ and *Λ*_0,*i*_ are the intrinsic phonon frequency and linewidth, respectively. *S*_0,*i*_ combines Raman phononic matrix element (*T*_p,*i*_), Raman electronic matrix element (*T*_e,*i*_) and *g*_*i*_, and it reads *S*_0,*i*_ = *T*_p,*i*_/(*T*_e,*i*_ ⋅ *g*_*i*_). *c*_*j*_, *w*_*j*_, and *ω*_*c*,*j*_ in the last term of Eq. (8) are area, linewidth, and center of the *j*th Lorentzian phonon, respectively. The parameter that determines the Fano asymmetry reads

9 $${q}_{i}(\omega )=-\frac{{S}_{i}(\omega )}{{\chi }_{e}^{^{\prime\prime} }(\omega )},$$

in which $1/{q}_{i}^{2}({\omega }_{0,i})$ describes the asymmetry in the phonon lineshape.

### DFT calculation

DFT calculations were performed for SrIrO_3_ using the mixed-basis pseudopotential method^57^ (Meyer, Elsässer, and Fähnle. *FORTRAN90 Program for Mixed-Basis Pseudopotential Calculations for Crystals*, unpublished). This method combines plane waves and local functions in the basis set, which allow an efficient description of more localized components of the valence states. In this study, we used plane waves up to a kinetic energy of 28 Ry, augmented by local functions of *s*, *p*, and *d* type at the Sr and Ir sites, respectively, and of *s* and *p* type at the O sites. Norm-conserving pseudopotentials were constructed following the description of Vanderbilt^58^, including the Sr-4*s*, Sr-4*p*, Ir-5*s*, Ir-5*p*, and O-2*s* semi-core states in the valence space. The local-density approximation in the parametrization of Perdew and Wang^59^ has been employed. Spin–orbit interaction is consistently incorporated in the DFT Hamiltonian by using a spinor formulation and by including spin–orbit components of the pseudopotentials^60^. For the orthorhombic structure of SrIrO_3_, we took the experimental lattice parameters of the film on the DyScO_3_ substrate^21^. Atomic positions were relaxed until the remaining forces were below 10^−3^ Ry/a.u. For the relaxation, it was sufficient to employ an orthorhombic 8 × 8 × 6 *k*-point mesh for BZ integration in conjunction with a Gaussian smearing of 0.1 eV. Due to the presence of flat hole-like bands close to the Fermi level, band structure and subsequent Fermi surfaces were determined with the tetrahedron method without any broadening, and with a very dense 32 × 32 × 24 *k*-point mesh.

The Fermi surface shown in Fig. 1b represents a top view for a (101) orientation of SrIrO_3_. The electron pockets at (±*π*/2, ±*π*/2) in 1-Ir BZ are a robust feature associated to steep bands, whereas the hole-like Fermi surface is an open surface arising from very flat bands and is very sensitive to structural details. Rough estimates for Fermi velocities *v*_F_ of holes and electrons have been derived from the band dispersion along high-symmetry directions. Effective band masses were estimated using the formula: *m*_b_ = (*ℏq*_F_)/*v*_F_, where *q*_F_ denotes the wavevector of the Fermi level crossing measured with respect to the pocket centers. This procedure gave average band masses for holes and electrons of 1.58*m*_e_ and 0.32*m*_e_, respectively.

## Supplementary information

Supplementary Information

Peer Review File

## Data Availability

The Raman data reported in this study have been deposited at the KIT Open, under the following identification number KITopen-ID: 1000119920. Any further relevant data are available from the authors on request to M.L.T. (matthieu.letacon@kit.edu).

## References

[1] Keimer B, Moore JE (2017). The physics of quantum materials. Nat. Phys..

[2] Tokura Y, Kawasaki M, Nagaosa N (2017). Emergent functions of quantum materials. Nat. Phys..

[3] Witczak-Krempa W, Chen G, Kim YB, Balents L (2014). Correlated quantum phenomena in the strong spin–orbit regime. Annu. Rev. Condens. Matter Phys..

[4] Sheehy DE, Schmalian J (2007). Quantum critical scaling in graphene. Phys. Rev. Lett..

[5] Assaad F, Herbut I (2013). Pinning the order: the nature of quantum criticality in the Hubbard model on honeycomb lattice. Phys. Rev. X.

[6] Moon E-G, Xu C, Kim YB, Balents L (2013). Non-Fermi-liquid and topological states with strong spin–orbit coupling. Phys. Rev. Lett..

[7] Kim BJ (2009). Phase-sensitive observation of a spin-orbital Mott state in Sr_2_ IrO_4_. Science.

[8] Bertinshaw J, Kim YK, Khaliullin G, Kim BJ (2019). Square lattice iridates. Annu. Rev. Condens. Matter Phys..

[9] Zhao JG (2008). High-pressure synthesis of orthorhombic SrIrO_3_ perovskite and its positive magnetoresistance. J. Appl. Phys..

[10] Cao G (2007). Non-Fermi-liquid behavior in nearly ferromagnetic SrIrO_3_ single crystals. Phys. Rev. B.

[11] Fujioka J, Okawa T, Yamamoto A, Tokura Y (2017). Correlated Dirac semimetallic state with unusual positive magnetoresistance in strain-free perovskite SrIrO_3_. Phys. Rev. B.

[12] Carter J-M, Shankar VV, Kee (2013). Theory of metal–insulator transition in the family of perovskite iridium oxides. Phys. Rev. B.

[13] Zeb MA, Kee H-Y (2012). Interplay between spin–orbit coupling and Hubbard interaction in SrIrO_3_ and related *P*_*b**n**m*_ perovskite oxides. Phys. Rev. B.

[14] Chen Y, Lu Y-M, Kee H-Y (2015). Topological crystalline metal in orthorhombic perovskite iridates. Nat. Commun..

[15] Nie YF (2015). Interplay of spin–orbit interactions, dimensionality, and octahedral rotations in semimetallic SrIrO_3_. Phys. Rev. Lett..

[16] Liu ZT (2016). Direct observation of the Dirac nodes lifting in semimetallic perovskite SrIrO_3_ thin films. Sci. Rep..

[17] Zhang H, Haule K, Vanderbilt D (2013). Effective *J* = 1/2 insulating state in Ruddlesden–Popper Iridates: an LDA.DMFT study. Phys. Rev. Lett..

[18] Biswas A, Kim K-S, Jeong YH (2014). Metal insulator transitions in perovskite SrIrO_3_ thin films. J. Appl. Phys..

[19] Matsuno J (2015). Engineering a spin–orbital magnetic insulator by tailoring superlattices. Phys. Rev. Lett..

[20] Zhang L, Pang B, Chen YB, Chen Y (2018). Review of spin–orbit coupled semimetal SrIrO_3_ in thin film form. Crit. Rev. Solid State Mater. Sci..

[21] Kleindienst KR (2018). Structural properties and anisotropic electronic transport in SrIrO_3_ films. Phys. Rev. B.

[22] Zhang L (2015). Tunable semimetallic state in compressive-strained SrIrO_3_ films revealed by transport behavior. Phys. Rev. B.

[23] Manca N (2018). Balanced electron–hole transport in spin-orbit semimetal SrIrO_3_ heterostructures. Phys. Rev. B.

[24] Fujioka J (2018). Charge dynamics and metal–insulator transition in perovskite SrIr_1−*x*_ Sn_*x*_ O_3_. J. Phys. Soc. Jpn..

[25] Moon SJ (2008). Dimensionality-controlled insulator–metal transition and correlated metallic state in 5*d* transition metal oxides Sr_*n*.1_Ir_*n*_O_3*n*.1_ (*n* = 1, 2, and *∞*). Phys. Rev. Lett..

[26] Blanchard PER (2014). Anomalous thermal expansion in orthorhombic perovskite SrIrO_3_: tnterplay between spin–orbit coupling and the crystal lattice. Phys. Rev. B.

[27] Lee D (2019). Engineering electrical property of Dirac semimetal perovskite SrIrO_3_ thin films by subtle changes in lattice structure. Appl. Phys. Express.

[28] Liu X (2017). Synthesis and electronic properties of Ruddlesden–Popper strontium iridate epitaxial thin films stabilized by control of growth kinetics. Phys. Rev. Mater..

[29] Groenendijk DJ (2017). Spin–orbit semimetal SrIrO_3_ in the two-dimensional limit. Phys. Rev. Lett..

[30] Devereaux TP, Hackl R (2007). Inelastic light scattering from correlated electrons. Rev. Mod. Phys..

[31] Devereaux TP, Einzel D (1995). Electronic Raman Scattering in superconductors as a probe of anisotropic electron pairing. Phys. Rev. B.

[32] Le Tacon M (2006). Two energy scales and two distinct quasiparticle dynamics in the superconducting state of underdoped cuprates. Nat. Phys..

[33] Cooper LN (1988). Gap anisotropy and phonon self-energy effects in single-crystal YBa_2_ Cu_3_ O_7−*δ*_. Phys. Rev. B.

[34] Bozovic I (1987). Optical measurements on oriented thin Ba_2_ Cu_3_ O_7−*δ*_ films: Lack of evidence for excitonic superconductivity. Phys. Rev. Lett..

[35] Virosztek A, Ruvalds J (1992). Raman spectrum of superconducting oxides. Phys. Rev. B.

[36] Varma, C. M. et al. Phenomenology of the normal state of Cu-O high-temperature superconductors. *Phys. Rev. Lett.* **63**, 1996 (1989). 10.1103/PhysRevLett.63.1996 10040734

[37] Sachdev, S. *Quantum Phase Transitions* (Cambridge Univ. Press, 1999).

[38] Zaanen J (2004). Superconductivity: why the temperature is high. Nature.

[39] Jaiswal AK (2019). Magnetotransport of SrIrO_3_ films on (110) DyScO_3_. AIP Adv..

[40] Hepting M (2015). Raman light scattering on ultra-thin films of LaNiO_3_ under compressive strain. Phys. B.

[41] Hepting M (2014). Tunable charge and spin order in PrNiO_3_ thin films and superlattices. Phys. Rev. Lett..

[42] Gallais Y (2013). Observation of incipient charge nematicity in $${\rm{Ba}}{({{\rm{Fe}}}_{1-X}{{\rm{Co}}}_{X})}_{2}{{\rm{As}}}_{2}$$. Phys. Rev. Lett..

[43] Kretzschmar F (2016). Critical spin fluctuations and the origin of nematic order in Ba(Fe_1−x_ Co_x_)_2_ As_2_. Nat. Phys..

[44] Fano U (1961). Effects of configuration interaction on intensities and phase shifts. Phys. Rev..

[45] Chen XK (1993). Oxygen-concentration dependence of the Raman continua in YBa_2_ Cu_3_ O_*y*_ single crystals. Phys. Rev. B.

[46] Götze W, Wölfle P (1972). Homogeneous dynamical conductivity of simple metals. Phys. Rev. B.

[47] Opel M (2000). Carrier relaxation, pseudogap, and superconducting gap in high-*T*_*c*_ cuprates: a Raman scattering study. Phys. Rev. B.

[48] Bruin JAN (2013). Similarity of scattering rates in metals showing T-linear resistivity. Science.

[49] Tytarenko A (2015). Direct observation of a Fermi liquid-like normal state in an iron-pnictide superconductor. Sci. Rep..

[50] Gurzhi RN (1959). Mutual electron correlations in metal optics. Sov. Phys. JETP.

[51] Chubukov AV, Maslov DL (2012). First-Matsubara-frequency rule in a Fermi liquid. I. Fermionic self-energy. Phys. Rev. B.

[52] Maslov DL, Chubukov AV (2012). First-Matsubara-frequency rule in a Fermi liquid. II. Optical conductivity and comparison to experiment. Phys. Rev. B.

[53] Stricker D (2014). Optical response of Sr_2_RuO_4_ reveals universal fermi-liquid scaling and quasiparticles beyond Landau theory. Phys. Rev. Lett..

[54] Nagel U (2012). Optical spectroscopy shows that the normal state of URu_2_ Si_2_ is an anomalous Fermi liquid. Proc. Natl Acad. Sci. USA.

[55] Sulewski PE (1988). Far-infrared absorptivity of UPt_3_. Phys. Rev. B.

[56] Watanabe H (2010). Microscopic study of a spin–orbit-induced Mott insulator in Ir oxides. Phys. Rev. Lett..

[57] Louie SG, Ho K-M, Cohen ML (1979). Self-consistent mixed-basis approach to the electronic structure of solids. Phys. Rev. B.

[58] Vanderbilt D (1985). Optimally smooth norm-conserving pseudopotentials. Phys. Rev. B.

[59] Perdew JP, Wang Y (1992). Accurate and simple analytic representation of the electron-gas correlation energy. Phys. Rev. B.

[60] Heid R, Bohnen K-P, Sklyadneva IY, Chulkov EV (2010). Effect of spin–orbit coupling on the electron–phonon interaction of the superconductors Pb and Tl. Phys. Rev. B.

