# Supplementary material for: Strange semimetal dynamics in SrIrO3
Source: Nat Commun. 2020 Aug 26;11:4270. doi: 10.1038/s41467-020-18092-6 (PMC7450065; doi:10.1038/s41467-020-18092-6)
Supplement: Supplementary file 1 — Supplementary Information [file 41467_2020_18092_MOESM1_ESM.pdf]

Strange semimetal dynamics in $SrIrO_3$

K. Sen *et al.*

Supplementary Note 1. Extraction of thin film Raman response using confocal Raman spectroscopy

Figure 1 illustrates the basic concept of Raman scattering in confocal geometry that allows to extract Raman response of ultrathin films. Figures 2(a)-(c) summarize the procedure to obtain Raman response $[\chi''(\omega)]$ of the 50 nm thick $SrIrO_3$ (SIO) film following this method in X′Y′ geometry at 20 K.

Notably, the Raman spectral background of the insulating $DyScO_3$ (DSO) substrate at Z= +15 μm in Fig. 2(a) is equivalent to the corresponding dark counts of the CCD, which indicating the absence of electronic Raman scattering in DSO. However, the finite spectral background at Z= 0 μm signifies the presence of a considerable electronic Raman scattering from the film. To confirm that such a finite spectral background is not the result of an experimental artefact, an insulating CeO_2 film (60 nm, grown on (001)-LSAT) was measured in the same way. In principal, the Raman spectral background should be zero for this insulating film since it has no unoccupied states at the Fermi level. Indeed, as shown in Fig. 3(a), the spectral backgrounds at Z= 0 and $Z = +15\,\mu$m are nearly equivalent, and they match reasonably well with the corresponding dark counts. Polarization selection rules for the measurement geometry allow only one phonon mode of E_g symmetry.[1] Indeed, we resolved that phonon mode, which is shown in Fig. 3(c).

Supplementary Note 2. Memory function formalism

The memory function formalism was first discussed in the context of optical conductivity by Götze and Wölfle.[2] Electronic Raman spectra were earlier analyzed within this formalism in ref.[3] The number of inelastically scattered photons per unit time is determined by the imaginary part of the total Raman response function $[\chi_e(\omega) = \chi'_e(\omega) + i\chi''_e(\omega)]$

$$\frac{dN(\omega)}{dt} \propto \left(1 + n_B(\omega)\right)\chi''_e(\omega)\,, \tag{1}$$

where $n_B(\omega)$ is the Bose function. $\chi_e(\omega)$ is a retarded function of the weighted density $\rho(\mathbf{q}) = \sum_{\mathbf{k}} \gamma_{\mathbf{k}} c^{\dagger}_{\mathbf{k}+\frac{\mathbf{q}}{2}} c_{\mathbf{k}-\frac{\mathbf{q}}{2}}$ in the limit $\mathbf{q} \to 0$. The form factor $\gamma_{\mathbf{k}}$ depends on the polarization geometry of the measurement. Since ρ is an Hermitian operator, it holds that

$$\chi_e(-\omega) = \chi^*_e(\omega)\,, \tag{2}$$

i.e. the real part of $\chi_e(\omega)$ is an even function while the imaginary part is an odd function. The memory function $[M(\omega)]$ parametrization of the Raman response function is

$$\chi_e(\omega) = \frac{M(\omega)}{\hbar\omega + M(\omega)}. \tag{3}$$

Thus, it holds that the memory function must obey

$$M(-\omega) = -M^*(\omega)\,. \tag{4}$$

Its real part is odd and the imaginary part is even. Thus, we can write

$$M(\omega) = \hbar\omega\lambda(\omega) + i\Gamma(\omega)\,,$$

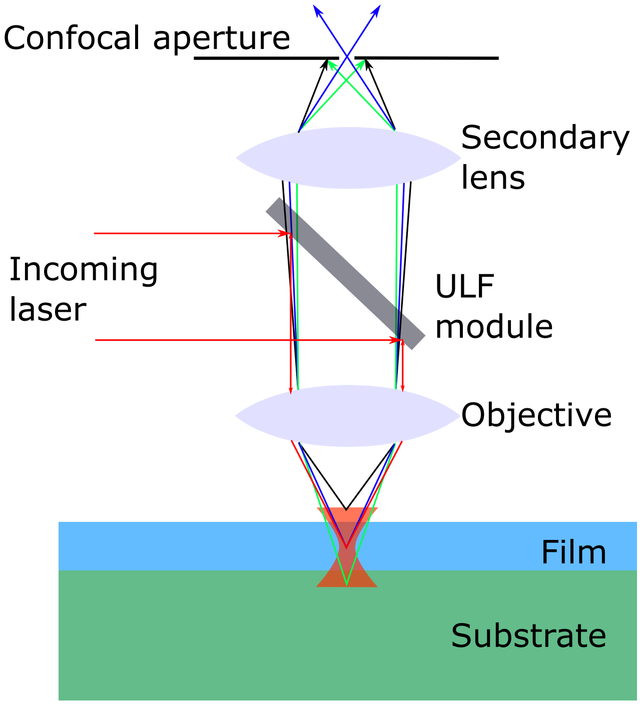

Figure 1: Confocal Raman scattering. The focal-point of microscope objective is placed on the substrate surface, which defines the reference position of the objective, *i.e.* Z= 0 μm, as mentioned in Methods of the main text. The incoming laser beam (red ray) is guided through the objective with the help of a elastic filter (ULF module). The inelastically scattered photons which pass through the secondary lens come from the thin film (blue ray), the substrate (green ray) and the vacuum (black ray). The elastic Rayleigh scattering is strongly reduced after the ULF module. In an ideal scenario, the optimum confocal aperture, which is placed right after the secondary lens, blocks the substrate- and vacuum-photons from entering into CCD. In reality, this configuration maximizes the thin film signal and strongly reduces the substrate signal. Subsequently, the pure substrate signal is recorded by placing the entire depth-of-focus of the microscope objective into the substrate at Z= +15 μm.

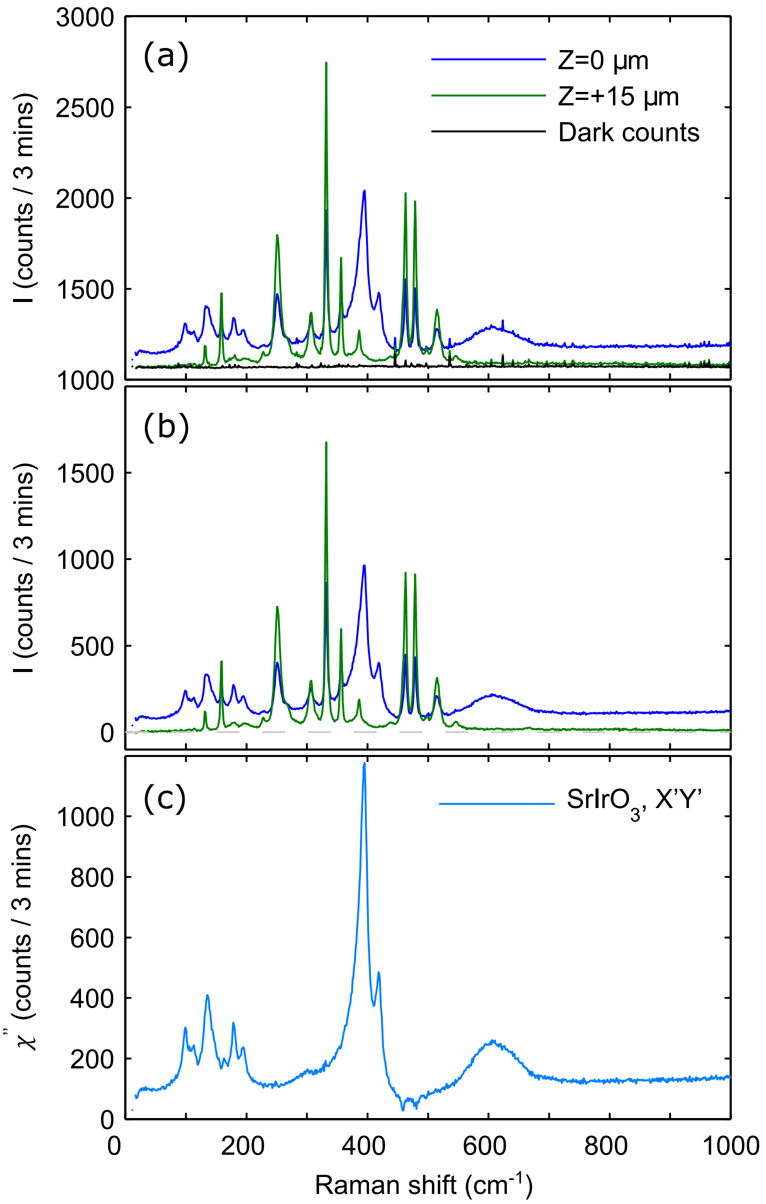

Figure 2: Raman response of a $SrIrO_3$ film on (101)-DSO in X′Y′ at 20 K. (a) Raman spectra recorded at the objective positions of Z= 0 (blue) and Z= +15 μm (green). The dark line corresponds to the dark counts of the CCD. The spectrum at Z= 0 μm consists the Raman signals of the film and the substrate. On contrary, the spectrum at Z= +15 μm has a pure substrate contribution. (b) The spectra after subtraction of the dark counts. (c) Raman signal of the film was extracted by subtracting the spectrum at Z= +15 μm from the one at Z= 0 μm using a proper scaling factor of ≈ 0.5. Subsequently, the Raman response $[\chi''(\omega)]$ of the film was obtained by normalizing the extracted film signal with the Bose factor and the response of the low-energy rejection filters.

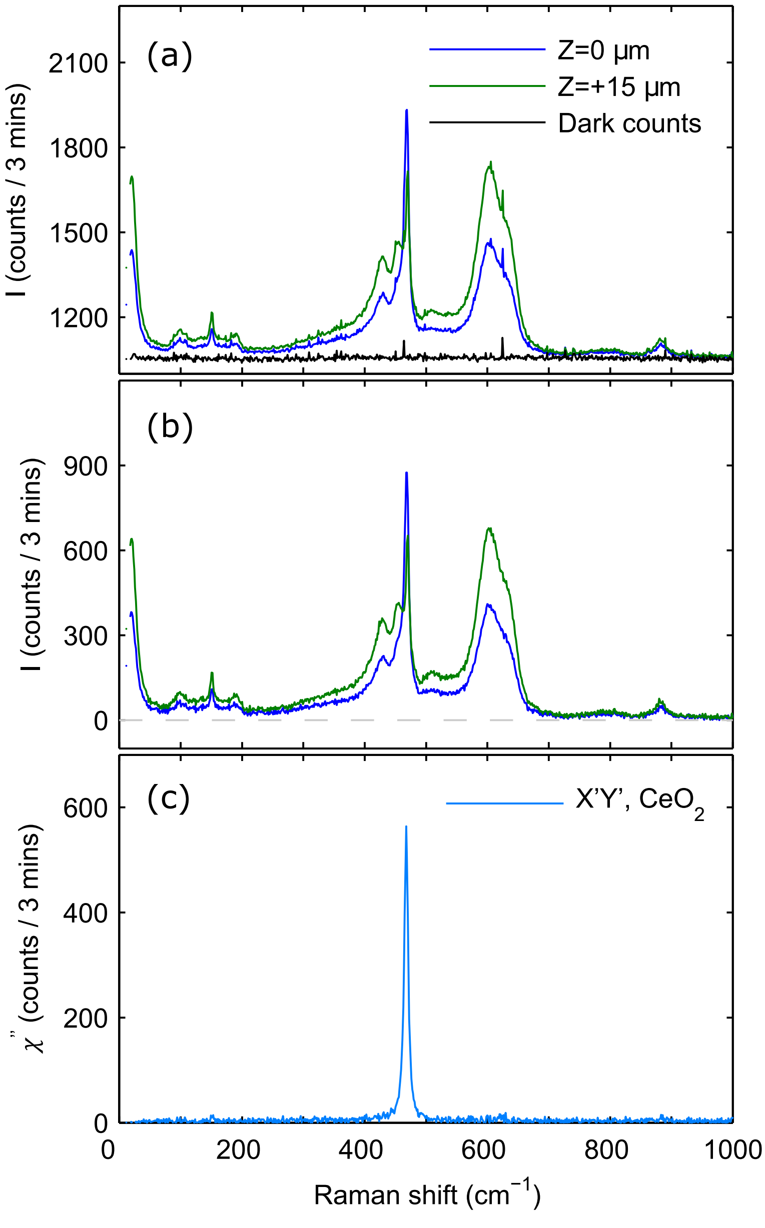

Figure 3: Raman response of a CeO_2 film on (001)-LSAT in X′Y′ at 60 K. (a) Raman spectra recorded at the objective positions of Z= 0 (blue) and Z= +15 μm (green). The dark line corresponds to the dark counts of the CCD. (b) The spectra after subtraction of the dark counts. (c) Raman response [$\chi''(\omega)$] of the film.

where both $\lambda(\omega)$ and $\Gamma(\omega)$ are real functions that are even under $\omega \to -\omega$. Here, $\Gamma(\omega)$ is the dynamic scattering rate of the charge carriers, and $1+\lambda(\omega)$ is the dynamic mass enhancement factor of the charge carriers. It follows

$$\chi_e''(\omega) = \frac{\hbar\omega\Gamma(\omega)}{\left[\hbar\omega\left(1+\lambda(\omega)\right)\right]^2 + \left[\Gamma(\omega)\right]^2}. \tag{5}$$

The complex memory function can always be generated from $\Gamma(\omega)$ using the Kramers-Kronig relation:

$$M(\omega) = -\frac{1}{\pi}\int_{-\infty}^{\infty} d\epsilon \frac{\Gamma(\epsilon)}{\hbar\omega + i0^+ - \epsilon}. \tag{6}$$

Since $\Gamma(\omega)$ is an even function this simplifies to

$$\lambda(\omega) = -\frac{2}{\pi}\lim_{\delta\to 0^+}\int_0^{\infty} d\epsilon \frac{\Gamma(\epsilon)\left(\hbar^2\omega^2 - \epsilon^2\right)}{\left(\hbar^2\omega^2 - \epsilon^2\right)^2 + \delta^2}. \tag{7}$$

In what follows we consider specific cases of $\Gamma(\omega, T)$ at finite temperatures for Fermi liquid (FL), non-Fermi liquid (NFL) and marginal Fermi liquid (MFL), and obtain the corresponding $\lambda(\omega, T)$ via Kramers-Kronig (KK) transformation.

Fermi liquid

In accordance with the expression for $\Gamma_{FL}(\omega, T)$ in eq. (4) in main text, we can write for memory function formalism that

$$\Gamma_{FL}(\omega, T) = \frac{g}{\hbar D}\left[(\hbar\omega)^2 + (\beta k_B T)^2\right]\phi\left(\omega/D\right), \tag{8}$$

where $\phi(t)$ is an appropriate cut-off function with a cut-off frequency of D, which ensures the convergence of the integrals. We use

$$\phi(t) = \frac{1}{1+t^2}. \tag{9}$$

Depending on the problem under consideration $\hbar D$ is of the order of band width of the relevant degrees of freedom or given by the Fermi energy (E_F). In the present case of SIO, $\hbar D$ is the band width for hole- and electron-like bands at E_F, and they are several hundreds of meV.

We can now perform KK transformation of $\Gamma_{FL}(\omega, T)$ in eq. (8) and obtain the following $\lambda_{FL}(\omega, T)$.

$$\lambda_{FL}(\omega, T) = g\left[1 - \frac{(\hbar\omega)^2 + (\beta k_B T)^2}{(\hbar D)^2}\right]\phi\left(\omega/D\right). \tag{10}$$

Non-Fermi liquid

The scattering rate $\Gamma_{NFL}(\omega, T)$ for non-Fermi liquid (NFL) can be written as

$$\Gamma_{NFL}(\omega, T) = \frac{g}{(\hbar D)^{2\alpha-1}}\left[(\hbar\omega)^2 + (\beta k_B T)^2\right]^{\alpha}\phi\left(\omega/D\right), \tag{11}$$

where α is the exponent for NFL, and can have the value of $\alpha < 1/2$ and $1/2 < \alpha \leq 1$. The KK transformation of $\Gamma_{NFL}(\omega, T)$ yields

$$\lambda_{NFL}(\omega, T) = \frac{g}{(\hbar D)^{2\alpha-1}}\left[-(\hbar D)^{2\alpha-1}\sec(\pi\alpha) + \left[(\hbar\omega)^2 + (\beta k_B T)^2\right]^{\frac{2\alpha-1}{2}}\tan(\pi\alpha)\right]\phi\left(\omega/D\right). \tag{12}$$

Marginal Fermi liquid

As already given in eq. (5) in main text, $\Gamma_{MFL}(\omega, T)$ for marginal Fermi liquid (MFL) reads

$$\Gamma_{MFL}(\omega, T) = g\sqrt{(\hbar\omega)^2 + (\beta k_B T)^2}\phi(\omega/D). \tag{13}$$

The KK transformation yields

$$\begin{aligned}\lambda_{MFL}(\omega, T) &= \frac{2g}{\pi}\phi\left(\frac{\omega}{D}\right)\left(\frac{\sqrt{1-\frac{(\beta k_B T)^2}{(\hbar D)^2}}}{2}\log\left(\frac{1+\sqrt{1-\frac{(\beta k_B T)^2}{(\hbar D)^2}}}{1-\sqrt{1-\frac{(\beta k_B T)^2}{(\hbar D)^2}}}\right)\right) \\ &\quad -\frac{2g}{\pi}\phi\left(\frac{\omega}{D}\right)\left(\sqrt{1+\frac{(\beta k_B T)^2}{(\hbar\omega)^2}}\operatorname{arccoth}\left(\sqrt{1+\frac{(\beta \mathrm{k_B T})^2}{(\hbar\omega)^2}}\right)\right). \end{aligned} \tag{14}$$

Effect of impurity scattering

Suppose we add a constant contribution Γ_{imp} to the scattering rate $\Gamma(\omega)$. It then follows that the corresponding effective mass $\lambda(\omega)$ is unchanged as the KK integral

$$\lambda_{\mathrm{c}}(\omega) = -\frac{2}{\pi}\lim_{\gamma\to 0^+}\int_0^\infty d\epsilon \frac{\Gamma_{imp}\left(\hbar^2\omega^2-\epsilon^2\right)}{\left(\hbar^2\omega^2-\epsilon^2\right)^2+\gamma^2} = 0.$$

To be consistent with our discussion of three types of charge dynamics we could include a weakly energy dependent cut-off function, *i.e.* have

$$\Gamma_{\mathrm{c}}(\omega) = \Gamma_{imp}\phi\left(\frac{\omega}{D}\right).$$

The KK transformation can be performed easily and it follows

$$\lambda_{\mathrm{c}}(\omega) = \frac{\Gamma_{imp}}{\hbar D}\phi\left(\frac{\omega}{D}\right). \tag{15}$$

Thus, the correction to the mass enhancement term is in the order $\Gamma_{imp}/\hbar D$. It can be safely neglected for SIO, as its $\hbar D$ for both hole- and electron-like bands amount to several hundreds of meV. It is enough to only add the additional scattering rate $\Gamma_c(\omega)$ to various $\Gamma(\omega, T)$ in eqs. (8), (11) and (13).

The quality of fit with various models

Here, we compare the quality of fit to our data with various $\Gamma(\omega, T)$ for FL, NFL and MFL. For representation, we only show fits to X′Y′ at 20 K, since the statistical noise in the data is considerably smaller than the counterpart in XY.

Fermi liquid fit. Fig. 4 shows global fit to $\chi''(\omega)$ in X′Y′ at 20 K with $\Gamma_{FL}(\omega, T)$ and $\lambda_{FL}(\omega, T)$ in eqs. (8) and (10) for Fermi liquid. The discrepancy between the fit and data is prominent, especially at low energy below $100\,\mathrm{cm}^{-1}$. This is because the electronic Raman response (χ''_e) for FL tends to be quasi-elastic-like response and strongly overestimates the data at low energy. In addition, we also notice that the FL response slightly underestimates the data towards high energy. The exactly same observations also hold for the data in XY. Therefore, the FL model is not an accurate description of the electronic Raman response in SIO.

Non-Fermi liquid fit. Fig. 5(a) shows global fits to $\chi''(\omega)$ in X′Y′ at 20 K with $\Gamma_{NFL}(\omega, T)$ and $\lambda_{NFL}(\omega, T)$ in eqs. (11) and (12) for non-Fermi liquid (NFL). In particular, we investigate the impact

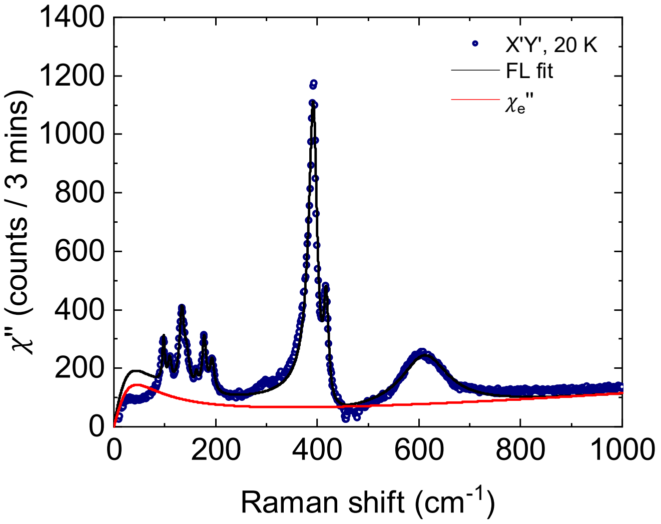

Figure 4: Global fit with Fermi liquid type electronic Raman response. Raman scattering response in X′Y′ at 20 K (symbols). The corresponding global fit (solid line in black) with the Fermi liquid (FL) scattering rate. Electronic Raman response (χ''_e) for FL is also shown (solid line in red). The corresponding fitting parameters are $g = 1.8$ and $\Gamma_{imp} = 14.6$ meV. The other fixed parameters are $\beta = 2\pi$ and cut-off energy $D = 250$ meV for the relevant hole-like band.

of the exponent α in eqs. (11) and (12) on the quality of global fit. The general observation is that the electronic Raman response at $< 100\,\text{cm}^{-1}$ increases with α. In an extreme case, when we set $\alpha = 1$ in eqs. (11) and (12) we approximately get back the Fermi liquid type response. In an agreement with our conclusion from Fig. 4, we again confirm that the Fermi liquid charge dynamics cannot represent the observed electronic Raman response in SIO. By inspecting the low-energy region below $100\,\text{cm}^{-1}$, we conclude that the optimum value of α would be $0.45 \leq \alpha \leq 0.6$. This is further manifested in the R^2 value in Fig. 5(b), which represents the goodness of fit. Therefore, our consideration of marginal Fermi liquid model ($\alpha = 0.5$) for the data analysis in main text is justified.

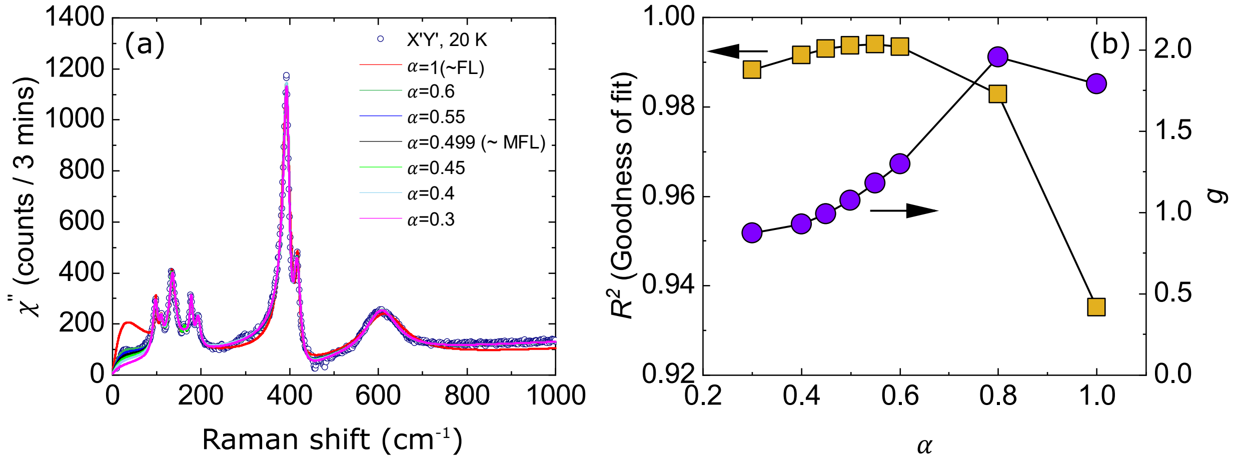

Figure 5: Global fit with non-Fermi liquid type electronic Raman response. (a) Raman scattering response in X′Y′ at 20 K (symbols). The corresponding global fits (solid lines) with non-Fermi liquid (NFL) type scattering rate as a function exponent α of eqs. (11) and (12). (b) The quality of fit is given in terms of R^2 values from the least square fits with different α. In addition, the corresponding fitting parameter g is also shown for different α at $\beta = 1.84$ and $\Gamma_{imp} = 12.6$ meV.

Strategy for fitting with marginal Fermi liquid model

The total scattering rate that was used to fit our data in X′Y′ and XY in main text is given by

$$\Gamma\left(\omega,T\right)=g\sqrt{\left(\hbar\omega\right)^{2}+\left(\beta k_{B}T\right)^{2}}\phi\left(\omega/D\right)+\Gamma_{imp}\phi\left(\omega/D\right). \tag{16}$$

The first term in eq. (16) is basically the Γ_{MFL} of eq. (13), and the second term arises from impurity scattering. We used the least squares fit to obtain the best fitting-curve by minimizing the sum squares of the offsets of the data points from the model. Such a minimization is a complex process in presence of several fitting parameters, such as g, β and Γ_{imp}, and their inter-dependence.

By definition β and Γ_{imp} are two temperature-independent quantities. We found that they affect the electronic Raman response at low energy ($\hbar\omega \lesssim k_BT$) in a similar way. Their optimum values were obtained (given in main text) by iterative fitting of the data at the lowest, highest and at an intermediate temperatures. Thereafter, β and Γ_{imp} were fixed at their optimum values for other temperatures. They are mentioned in main text. On the other hand, the parameter g was always set free.

Uncertainty and maximum proportional error

We obtained the uncertainty for β and Γ_{imp} by allowing the corresponding parameters to vary around their optimum values at all temperatures to attain best fits. Finally, the uncertainty was calculated from the minimum and maximum values of β and Γ_{imp}. In X′Y′, the uncertainty of β and Γ_{imp} are $\Delta\beta = 0.3$ and $\Delta\Gamma_{imp} = 16\,\mathrm{cm}^{-1}$, respectively. They are 0.5 and $40\,\mathrm{cm}^{-1}$ in XY, respectively. On the other hand, standard error calculated from least squares fit represents the uncertainty (Δg) of g parameter very well. The corresponding errorbar is shown in Fig. 7(a).

Maximum proportional error for scattering rate in static limit. The scattering rate in static limit amounts to $\Gamma_0 = \Gamma(\omega \to 0, T) = g\beta T + \Gamma_{imp}$, as shown in Fig. 7(b). The uncertainty ($\Delta\Gamma_0$) in determination of Γ_0 can be calculated using the maximum proportional error scheme, following eq. (17).

$$\Delta\Gamma_0=\sqrt{\beta^2T^2(\Delta g)^2+g^2T^2(\Delta\beta)^2+(\Delta\Gamma_{imp})^2} \tag{17}$$

Uncertainty in determination of inverse inelastic quasiparticle time ($\hbar\tau^{-1}_{\text{inel.}}$). As defined in main text, $\hbar\tau^{-1}_{\text{inel.}} = (\Gamma_0 - \Gamma_{imp})/(1+\lambda_0) = g\beta T/(1+\lambda_0)$. Therefore, the uncertainty of $\hbar\tau^{-1}_{\text{inel.}}$ reads

$$\Delta\left(\hbar\tau^{-1}_{\text{inel.}}\right)=\hbar\tau^{-1}_{\text{inel.}}\sqrt{\left(\frac{\Delta g}{g}\right)^2+\left(\frac{\Delta\beta}{\beta}\right)^2+\left(\frac{\Delta(1+\lambda_0)}{1+\lambda_0}\right)^2}, \tag{18}$$

where the uncertainty of $(1+\lambda_0)$ was calculated from the variation of $(1+\lambda_0)$ in the resultant fits by varying β and Γ_{imp} within their uncertainty (see Fig. 7(c)).

Uncertainty in determination of DC mobility (μ). As discussed in main text, the DC (static) mobility of charge carriers is defined by $\mu = e\tau_0/m_0^* = e\hbar/m_b\Gamma_0$. Thereby, we can easily calculate the mobility uncertainty ($\Delta\mu$) using eq. (19).

$$\Delta\mu=\mu\frac{\Delta\Gamma_0}{\Gamma_0} \tag{19}$$

Cut-off energy and its effect on $\boldsymbol{\Gamma(\omega,\mathrm{T})}$ and $\boldsymbol{\lambda(\omega,\mathrm{T})}$

Electronic Raman scattering in X′Y′ geometry probes the hole-pockets which arise from the hole-like bands that are extending along Γ-S, as show in Fig. 10. The corresponding band width amounts to $\sim 250\,\mathrm{meV}$, which sets the cut-off energy ($\hbar D$) for memory function formalism in X′Y′. Electronic

Raman response in XY geometry arises from the electron-like bands at high-symmetry U and T points in the band structure shown in Fig. 10. Their band widths of $\sim 550\,\mathrm{meV}$ set the energy scale of $\hbar D$ in XY.

Notably, the flat hole-like bands are rather narrow and their bandwidths are very sensitive to the relaxation of internal structural parameters. We tested the impact of $\hbar D$ on $\Gamma(\omega, T)$ and $\lambda(\omega, T)$ in X′Y′ by setting its value from 250 to 300 meV. The results are shown in Fig. 6. We found that the static scattering rate $\Gamma_0 = \Gamma(\omega \to 0)$ is unaffected. However, the mass enhancement factor $(1 + \lambda_0)$ in the dc limit increases by 4.5 %. At $1000\,\mathrm{cm}^{-1}$, Γ and $1 + \lambda$ increases by 8 and 10 %, respectively.

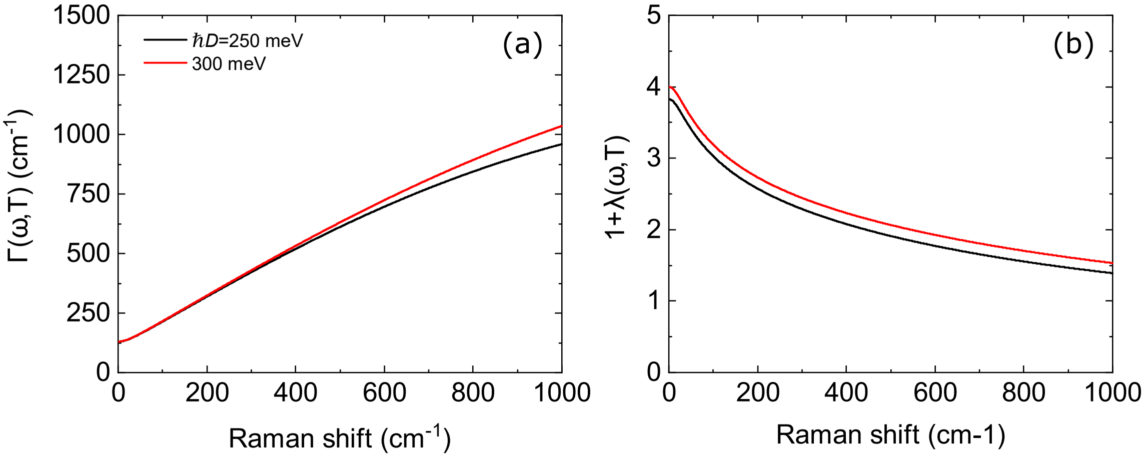

Figure 6: The impact of $\hbar$D on Γ and $1 + \lambda$. (a) Scattering rate $\Gamma(\omega, T)$ and (b) mass enhancement factor $1 + \lambda(\omega, T)$ in X′Y′ at 20 K.

g, Γ_0 and $1 + \lambda_0$ from memory function formalism for MFL

Fig. 7 summarizes the temperature-dependent coupling coefficient (g), static scattering rate (Γ_0) and mass enhancement factor ($1 + \lambda_0$) for conduction holes (X′Y′) and electrons (XY).

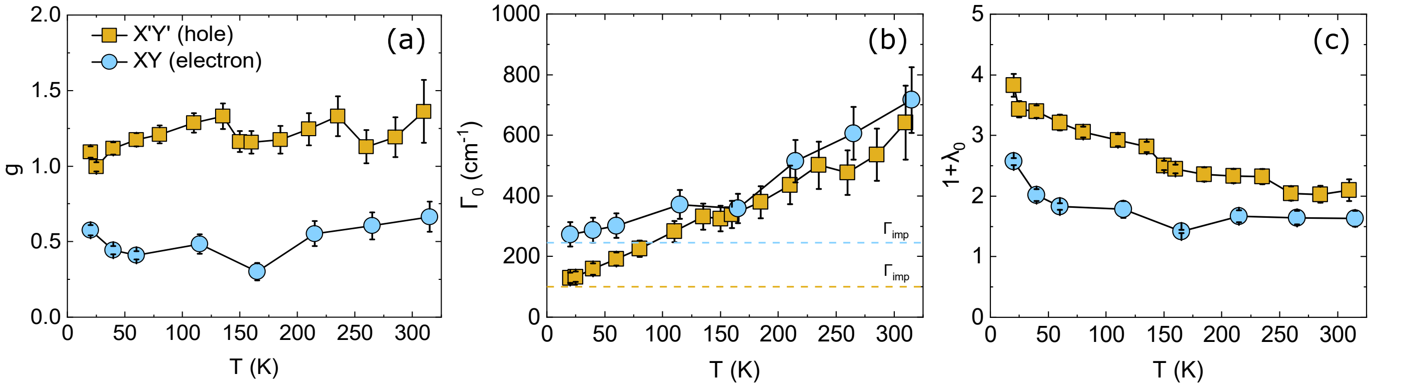

Figure 7: Results from memory function formalism for MFL. (a) The coupling coefficient g as a function of temperature. The corresponding errorbar indicates standard error from the least squares fits. (b) The scattering rate at the static limit, $\Gamma_0 = \Gamma(\omega \to 0, T) = g\beta k_B T + \Gamma_{imp}$. The error bar of Γ_0 is obtained from maximum proportional error scheme. (c) The mass enhancement factor $[m^*/m_b(\omega \to 0, T)]$ at static limit, $1 + \lambda_0 = 1 + \lambda(\omega \to 0, T)$. The corresponding errorbar was calculated from the variation of $1 + \lambda_0$ in the resultant fits by varying β and Γ_{imp} within their uncertainty.

Supplementary Note 3. Simulation of Hall resistance vs. applied magnetic field

The Hall resistance (R_{XY}) for two parallel channels of hole- and electron-like charge carriers can be expressed as[4]

$$\frac{tR_{xy}}{B} = \frac{1}{e}\frac{n_h\mu_h^2 - n_e\mu_e^2 + (n_h - n_e)(\mu_h\mu_e B)^2}{\sigma_0^2/e^2 + (n_h - n_e)^2(\mu_h\mu_e B)^2}, \tag{20}$$

where

$$\sigma_0 = \sigma_h + \sigma_e = e(n_h\mu_h + n_e\mu_e). \tag{21}$$

Here, e, t and B are electronic charge, film thickness and applied magnetic field, respectively. n_h (n_e) and μ_h (μ_e) are density and mobility of conduction holes (electrons), respectively.

Following eqs. (20) and (21), we simulated R_{xy} as a function of B for the 50 nm thick $SrIrO_3$ film using the obtained mobility of conduction holes and electrons, as summarized in Table I in the main text. We considered the charge densities: $n_e = 3.0 \times 10^{26}\,\mathrm{m}^{-3}$ and $n_h = 1.5{\times}n_e$ at 20 K (they increase by 6 % at room temperature), as discussed in the main text. R_{xy} in Fig. 8 appears to be linear as a function of B with negative gradient, which indicating electron-dominated Hall resistance, as found in several experiments.[4,5,6] We further revealed that R_{xy} becomes non-linear as a function of B only above a large magnetic field of $B > 150$ T for the given charge transport parameters.

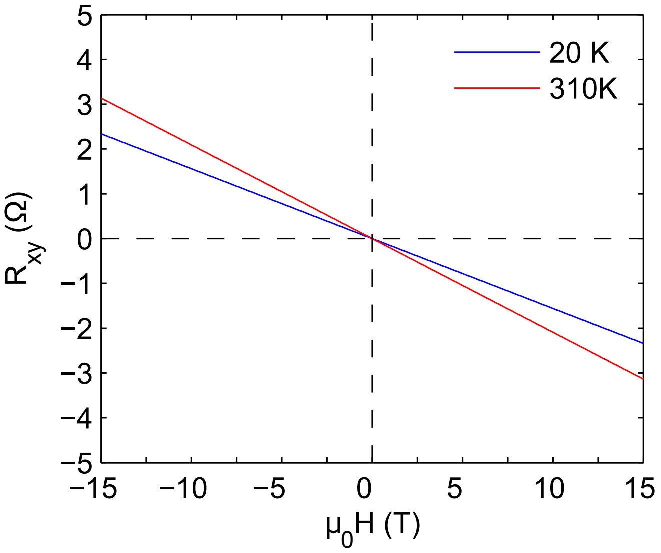

Figure 8: Hall resistance. Simulated Hall resistance (R_{xy}) as a function of applied magnetic field (B) at 20 and 310 K for 50 nm thick SIO film.

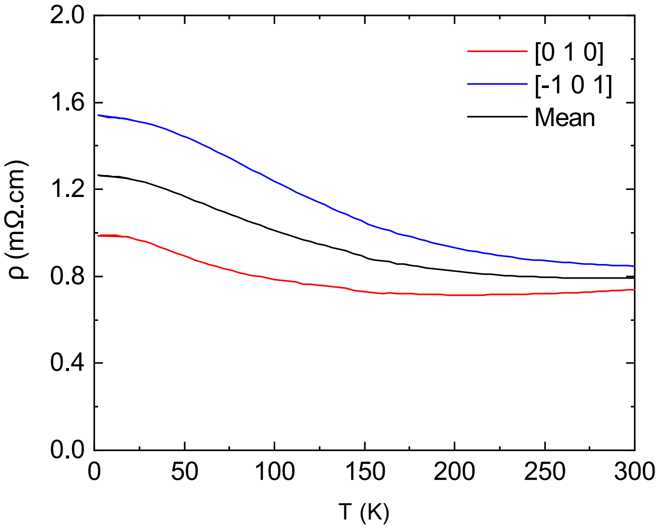

Figure 9: DC resistivity. DC resistivity of our SIO film for the two orthogonal directions of the DSO substrate.

Supplementary Note 4. First-principle calculations

For the orthorhombic structure of SIO, following ref.,[7] the *Pbnm* setting was assumed, which is a non-standard, but equivalent setting of *Pnma*. Taking the experimental lattice constants of the film on the $DyScO_3$ substrate,[6] the internal structural parameters were relaxed. Values are shown in Table 1. We found only small deviations from the bulk values reported by Blanchard *et al.*,[7] indicating that the structural modifications induced by the substrate are rather small.

The band structure is shown in Fig. 10 along various high-symmetry lines of the Brillouin zone. The presence of very flat hole like bands close to the Fermi level results in a step like behavior of the density of states, the application of a broadening scheme tends to put the Fermi level into a wrong position. This was the reason, why band structure and subsequent Fermi surfaces were determined with the tetrahedron method without any broadening and with a very dense $32 \times 32 \times 24$ k-point mesh.

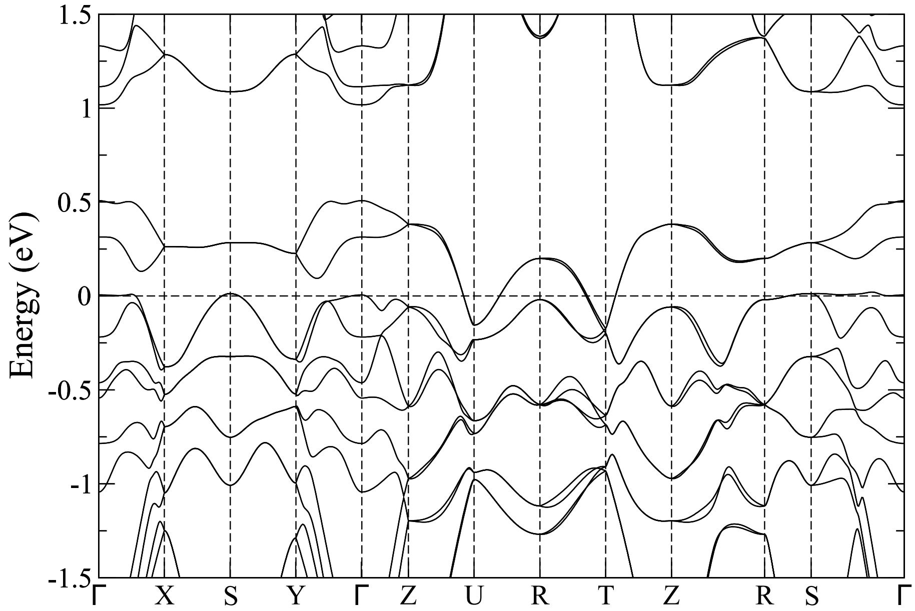

Figure 10: Band structure. Electronic band structure of the $SrIrO_3$ thin film for the theoretically relaxed unit cell, as described in Table 1.

The Fermi surface shown in Fig. 1(b) of the main text is derived from this band structure and

represents a top view for the $(1\,0\,1)_{Pnma}$ orientation of SIO. The electron pockets at $(\pm\pi/2, \pm\pi/2)$ in 1-Ir BZ (for the pseudocubic unit cell shown in Fig. 1(a) of main text) arise from steep bands around the high symmetry U and T points of the orthorhombic BZ. The hole-like Fermi surface appearing at $(0, \pm\pi)$, $(\pm\pi, 0)$ and $(\pm\pi, \pm\pi)$ in the 1-Ir BZ is an open surface which derives from bands at S and Γ. As these bands barely cross the Fermi level, the shape of the hole-like Fermi surface is very sensitive to structural details, while the electron pockets are a rather robust feature of the band structure.

	a	b	c	Sr-x	Sr-y	O1-x	O1-y	O2-x	O2-y	O2-z
Th.	5.61	5.59	7.92	-0.0068	0.4633	0.0752	0.0187	0.2120	0.2884	-0.0393
Exp.	5.5887	5.5725	7.8841	-0.0071	0.4644	0.0764	0.0093	0.2110	0.2879	-0.0373

Table 1: Theoretically relaxed internal parameters for $SrIrO_3$ for the experimental lattice constants[6] determined for the film on the $DyScO_3$ substrate (Th.), compared with experimental data for bulk $SrIrO_3$ (Exp.) at 3 K reported by Blanchard *et al.*[7] In the orthorhombic space group of *Pbnm*, Sr and O1 are on 4c sites $(x\,y\,\frac{1}{4})$, Ir on 4a sites $(0\,0\,0)$, and O2 on 8d sites $(x\,y\,z)$.

Supplementary Note 5. Contribution of acoustical phonons to the electronic scattering rate

The contribution of acoustical phonons to the electronic (static) scattering rate in metals can be evaluated from the Bloch-Grüneisen theory (BGT),[8,9] and is given by:

$$\hbar\tau_{\mathrm{ep}}^{-1} = 2\lambda_{\mathrm{ep}} \frac{k_B T^5}{\theta_D^4} \int_0^{\theta_D/T} \frac{x^5}{\cosh(x) - 1} dx, \tag{22}$$

where θ_D is the Debye temperature and λ_{ep} is the dimensionless electron-phonon interaction. At high temperature $T \gg \theta_D$ holds $\hbar\tau_{\mathrm{ep}}^{-1} \approx \lambda_{\mathrm{ep}} k_B T$. On the other hand, at low temperatures $T \ll \theta_D$ one finds

$$\hbar\tau_{\mathrm{ep}}^{-1} \approx 480\zeta(5)\,\lambda_{\mathrm{ep}} k_B T^5/\theta_D^4 \tag{23}$$

, where ζ is the zeta function. The temperature dependence of the scattering rate is shown in the left panel of Fig. 11. Here we see that a true linear behavior with correct slope can be seen for $T \geq \theta_D$.

To identify the temperature range in which the cross-over between the T^5 and linear-T behavior occurs, we show in the right panel of the figure $\hbar\tau_{\mathrm{ep}}^{-1}$ divided by the leading low-T behavior of eq. (23).

We observe that the behavior of eq. (23) is correct up to $T \approx \theta_D/10$ (this contrasts with the value $\theta_D/50$ sometimes found in the literature[8]). More importantly, the full temperature dependence of the left panel shows that strong deviations from the linear behavior are clearly visible at temperatures up to $\theta_D/5$.

In $SrIrO_3$ $\theta_D \sim 800K$ (which is given by the highest calculated phonon frequency), so it is expected that the cross-over between the low-temperature and the high-temperature regime will take place around $100K$. The electronic scattering rate can be directly extracted from the raw data as the inverse of the slope of the electronic Raman response. As shown in Fig. 12, this displays a linear behavior over the whole temperature range. To estimate the influence of electron-phonon scattering, we can compare it with the prediction of the BGT, placing ourselves in the (unrealistic) limit in which the scattering at room temperature is entirely caused by acoustical phonon (on top of the temperature independent residual scattering from e.g. lattice defects).

We can clearly see that over a wide range of temperatures, phonon scattering cannot account for the observed behavior (so in the more realistic case where it would only account for a fraction of

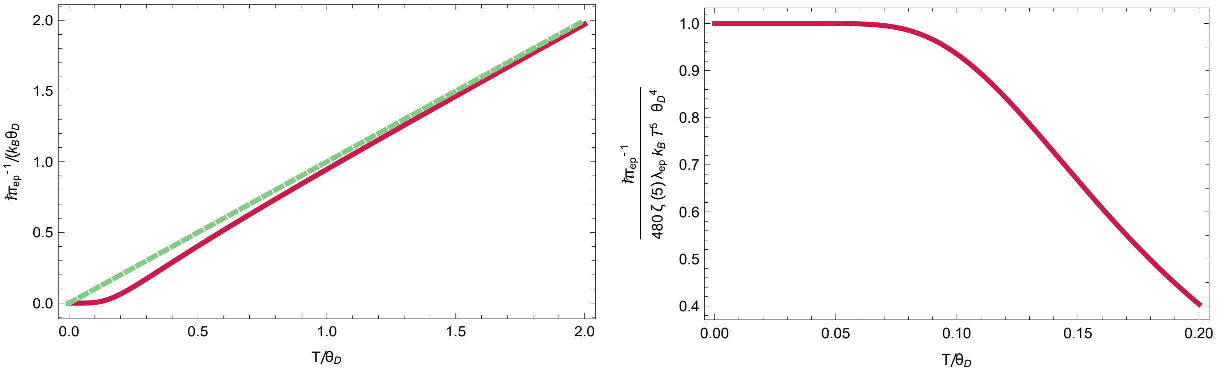

Figure 11: Bloch-Grüneisen scattering. Left panel: Temperature dependence of the Bloch-Grüneisen scattering rate and hence of the resistivity due to electron-phonon scattering. Right panel: scattering rate divided by the leading low-T behavior $\tau_{ep}^{-1} \propto T^5$, demonstrating that it is valid for $T < \theta_D/10$.

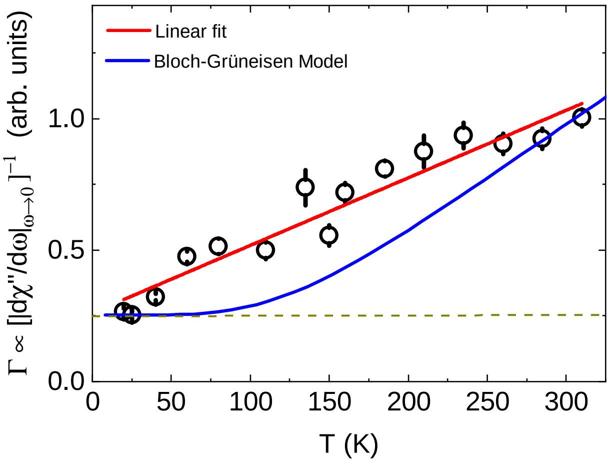

Figure 12: Low-energy slope of the Raman response in X'Y' (hole carriers).

the scattering at room temperature, it would generally be negligible). Alongside the presence of a broad continuum at high energies, this strongly suggests that scattering from phonons, if present, do not play a dominant role in shaping the electronic Raman response. As on the other hand, the electronic Raman response can be satisfactorily reproduced using the phenomenology of the marginal Fermi liquid, no contribution from the acoustical phonon scattering to the total scattering rate was considered.

References

[1] Weber, W. H. and Hass, K. C. and McBride, J. R. Raman study of CeO_2. Second-order scattering, lattice dynamics, and particle-size effects. *Phys. Rev. B* **48**, 178 (1993).

[2] Götze, W. and Wölfle, P. Homogeneous dynamical conductivity of simple metals. *Phys. Rev. B* **6**, 1226 (1972).

[3] Opel, M. *et al.* Carrier relaxation, pseudogap, and superconducting gap in high-T_c cuprates: A Raman scattering study. *Phys. Rev. B* **61**, 9752 (2000).

[4] Manca, Nicola *et al.* Balanced electron-hole transport in spin-orbit semimetal $SrIrO_3$ heterostructures. *Phys. Rev. B* **97**, 081105(R) (2018).

[5] Nie, Y. F. *et al.* Interplay of Spin-Orbit Interactions, Dimensionality, and Octahedral Rotations in Semimetallic $SrIrO_3$. *Phys. Rev. Lett.* **114**, 016401 (2015).

[6] Kleindienst, K. R. *et al.* Structural properties and anisotropic electronic transport in $SrIrO_3$ films. *Phys. Rev. B* **98**, 115113 (2018).

[7] Blanchard, Peter E. R. *et al.* Anomalous thermal expansion in orthorhombic perovskite $SrIrO_3$: Interplay between spin-orbit coupling and the crystal lattice. *Phys. Rev. B* **89**, 214106 (2014).

[8] Bass, Jack and Pratt, William P. and Schroeder, Peter A. The temperature-dependent electrical resistivities of the alkali metals. *Rev. Mod. Phys.* **62**, 645 (1990).

[9] Levchenko, Alex and Schmalian, Joerg Transport properties of strongly coupled electron-phonon liquids. *Annals of Physics* **419**, 168218 (2020).
